# Systematic importance of morphological features of pollen grains of species from *Erica* (Ericaceae) genus

**DOI:** 10.1371/journal.pone.0204557

**Published:** 2018-10-24

**Authors:** Dorota Wrońska-Pilarek, Piotr Szkudlarz, Jan Bocianowski

**Affiliations:** 1 Department of Forest Botany, Poznan University of Life Sciences, Poznań, Poland; 2 Department of Plant Taxonomy, Faculty of Biology, Adam Mickiewicz University, Poznań, Poland; 3 Department Mathematical and Statistical Methods, Poznan University of Life Sciences, Poznań, Poland; Universita degli Studi di Perugia, ITALY

## Abstract

The *Erica* genus has as yet not been investigated satisfactorily in terms of palynology. Its complicated taxonomic system, large number of species, as well as its extensive but disrupted range of occurrence, all contribute to the fact that few researchers have undertaken investigations of this species. It was assumed that the research results would be representative thanks to a complex comparative analysis of all diagnostical, morphological pollen features performed on properly selected plant material, representing the most important distinguished intrageneric taxons at the present time (45 species from all five subgenera and 22 sections), both discriminated pollen dispersal units (tetrads and monads) as well as the main centres of genus occurrence and diversification (species from Europe, the Republic of South Africa (RSA) and Madagascar). The study revealed that the diagnostic features of the pollen grains studied were: pollen dispersal unit, exine ornamentation, P/E ratio, tetrad diameter (D) and length of polar axis (P). On the basis of these traits, 14 *Erica* species (six creating monads and eight—tetrads) were distinguished which, in the case of pollen features, constitutes a significant number. Other heaths created small groups, usually containing two or three species, but up to seven species. The present study, based on the highest number of *Erica* species (45) analysed so far, corroborated the view that an examination of palynological features may assist in clarifying classification systems for the large and taxonomically very difficult *Erica* genus, in particular, at the level of the subgenus and section, but also at species level. The results obtained indicate the need to continue palynological investigations on the *Erica* genus.

## Introduction

The family Ericaceae comprises eight subfamilies, approximately 125 genera and 4100 species [1]. Recently, a revised phylogenetic classification of the Ericaceae family was proposed by [2], which recognizes eight subfamilies and 20 tribes, and identifies *Cyrilla* (Cyrillaceae) as a sister to the Ericaceae. However, tribal and generic circumscriptions thus far remain ambiguous [e.g. 3–5]. The *Erica* genus belongs to the subfamily Ericoideae Link, which is divided into five tribes: *Bejarieae*, *Phyllodoceae*, *Ericeae*, *Empetreae*, and *Rhodoreae*, with only 19 genera and approximately 1780 species [1].

The *Erica* genus, alongside the *Rhododendron* genus, belongs to the most numerous genera in the Ericaceae family and, according to the most recent data, comprises 865 species [6, 7]. This very numerous and strongly diversified genus presents multiple problems from the point of view of its taxonomy. One of the taxonomists [8] put forward a completely different concept from earlier approaches of the *Erica* genus in which the basic feature was the structure of the stamens. He divided the genus into 4 subgenera and, within their boundary, into 49 sections. The basis of the latest taxonomic system of the *Erica* genus [9] was the structure of the crown and calyx. They distinguished 5 subgenera divided into 41 sections. This study referred only to taxons of the Capensis area and did not include the entire genus. In a further revision for the entire Capensis area 605 species was distinguished [10]. However, his study did not put forward any new proposal concerning the internal division of the genus. The author verified and put in order numerous synonyms, including species described from the publication of Flora Capensis but leaving the previously proposed [9] species numeration. A successive, hitherto unfinished, revision of the *Erica* genus was carried out in 2002 and 2005 [11, 12]. He included the so-called minor genera in the *Erica* genus; however, he has as yet failed to allocate places in the subgenera and sections to these taxons [13–19]. In his proposal, he left intact the concept of the earlier proposed [9] genus division.

The range of the occurrence of representatives of the *Erica* genus extends from northern areas of Europe to southern edges of Africa. Towards the East, the genus stretches to central Turkey and the south-western part of the Arab Peninsula. Taxon density in the above-described area is very uneven [6]. In Europe, 20 (21) species of heathers occur [6, 20]. In tropical Africa, heathers can be found in small centres with 2 to 22 species, always associated with mountainous regions [6]. A separate centre of occurrence is found in Madagascar with 35 species [21]. The area characterised by the greatest species diversity and abundance is South Africa, in particular in the Cape Floristic Region, where up to 760 species from the *Erica* genus can be found.

The pollen morphology of some *Erica* taxa has been studied fragmentally for taxonomic purposes and most of these studies have referred to the entire Ericaceae family or to the Ericales order (e.g. [6, 22–28]) or constituted part of regional flora studies (e.g. [29–34]). Only two publications have been devoted exclusively to the pollen morphology of the *Erica* genus [35, 36].

On the basis of the palynological investigations conducted so far, it is known that the dispersal units of *Erica* pollen grains are tetrads (tetrahedral tetrads), and rarely monads [28; 36–39].

The pollen grains of *Erica* are 3 (4) colporate or 3 (4) colporoidate [28, 37]. The pollen grains are small or medium-sized, and oblate to prolate in shape. Exine ornamentation is changeable and can be psilate, gemmate-psilate, rugulate-psilate, verrucate-psilate, granulate, verrucate, microgranulate, microgammate or microstriate [28, 35, 36]. Other authors [37–39] also reported that fossulate exine ornamentation frequently occurred.

The diagnostic features of *Erica* pollen grains are: dispersal unit of pollen grains, pollen and tetrad size, pollen shape and exine ornamentation [6, 28, 35, 36, 38, 39].

The study presented was undertaken to analyse in a complex manner the relationships among the taxa within the *Erica* genus, based on the palynological features, as well as to discuss the taxonomic significance with reference to the current classification of this genus.

One of the strengths of this study is that it used the largest number of species (45) so far and these species derived from an extensive area of large *Erica* genus (Table 1). Monographic investigations based on such a large number of species have hitherto not been the object of palynological studies. Until now, the most extensive research comprised 23 *Erica* species [36], of which only five (*E*. *arborea*, *E*. *axillaris*, *E*. *cinerea*, *E*. *nabea*, *E*. *tetralix*) were also examined in the study presented. Previously, fossil pollen grains of 17 European *Erica* species was described [35], of which five (*E*. *arborea*, *E*. *australis*, *E*. *cinerea*, *E*. *erigena*, *E*. *tetralix*) also appeared in the present study. In addition, the interspecific variability of the pollen grains of the *Erica* taxa under investigation has not yet been comprehensively analysed. Another advantage of this work is the fact that it examined endemic species from Madagascar not investigated thus far and earlier included in the *Philippia* genus. This allowed a comparison of the pollen grains representing this centre of occurrence with species representing the so-called minor genera, as well as the heather species *sensu stricto*.

**Table 1:** List of localities of the *Erica* studied species.

No	Subgenus	Section	Species	Localities	Position	Collector, herbarium
1.	*Syringodea*	*Gigandra*	*E*. *coccinea* L.	RSA, Cape Agulhas	S 34°49’05” E 20°01’06”	SWO 143, POZG
2.	*Syringodea*	*Gigandra*	*E*. *plukenetii* subsp. *plukenetii* L.	RSA, Ezeljacht, 173 m a.s.l.	S 33°31’11” E 18°27’55”	SWO 40, POZG
3.	*Syringodea*	*Pleurocallis*	*E*. *regia*Bartl. Subbsp regia var. Casta	RSA, Cocsravier near Viljoenshof, 149 m a.s.l.	S 34°40’30” E 19°38’30”	SWO 135, POZG
4.	*Syringodea*	*Pleurocallis*	*E*. *vestita* Thunb.	RSA, Jonaskop, 540 m a.s.l.	S 33°55’18” E 19°30’58”	SWO 172, POZG
5.	*Syringodea*	*Evanthe*	*E*. *curviflora* L.	RSA, Pilaarkop	S 34°05’11” E 19°51’10”	SWO 148, POZG
6.	*Syringodea*	*Evanthe*	*E*. *discolor* Andrews	RSA, road from Keurboomsriver to Kruisvallei	S 33°56’27” E 23°18’47”	SWO 204, POZG
7.	*Stellanthe*	*Callista*	*E*. *denticulata* L.	RSA, Theevaterklof	S 33°58’09” E 19°10’015”	SWO 50, POZG
8.	*Stellanthe*	*Callista*	*E*. *fastigiata* L.	RSA, vicinity of Caledon,	S 34°13′48″ E 19°25′42″	SWO 79A, POZG
9.	*Euerica*	*Ephebus*	*E*. *peziza* Lodd.	RSA, Stormsvlei	S 34°03’52” E 20°04’58”	SWO 190, POZG
10.	*Euerica*	*Ephebus*	*E*. *parviflora* L.	RSA, Theevaterklof	S 33°58’40” E19°10’30,9”	SWO 47, POZG
11.	*Euerica*	*Ephebus*	*E*. *hirtiflora* Curtis	RSA, Kirstenbosch, slope of the Tablle Montain	S 33°59’16” E 18°25’51,5”	SWO 12, POZG
12.	*Euerica*	Ephebus	E. caffra L.	RSA, Swartberg Pass, 1350 m a.s.l.	S 33°21’26”, E 22°03’28”	SWO 221, POZG
13.	*Euerica*	Orophanes	E. tenella Andrews	RSA, Boskloof	S 34°24’30” E 19°40’22”	SWO 108, POZG
14.	*Euerica*	*Orophanes*	*E*. *trichophylla* Benth.	RSA, Pilaarkop,	S 34°05’11” E 19°51’10”	SWO 167, POZG
15.	*Euerica*	*Pachysa*	*E*. *zwartbergensis* Bolus	RSA, Swartberg Pass, 1117 m a.s.l.	S 33°21’42” E 22°05’14”	SWO 214, POZG
16.	*Euerica*	*Arsace*	*E*. *hispidula* L.	RSA, Boskloof	S 34°24’30” E 19°40’22”	SWO 118, POZG
17.	*Euerica*	*Arsace*	*E*. *arborea* L.	Spain, Lugo	N 42°23’26” W 007°12’20,1”	Szkudlarz P, POZ
18.	*Euerica*	*Callicodon*	*E*. *erigena* R.Ross	Spain, Boveda	N 42°38’34” W 007°28’37,3”	Szkudlarz P, POZ
19.	*Euerica*	*Pyronium*	*E*. *umbellate* L.	Spain, Lugo	N 42°23’26” W 007°12’20,1”	Szkudlarz P, POZ
20.	*Euerica*	*Tylospora*	*E*. *australis* L.	Spain, Boveda	N 42°38’34” W 007°28’37,3”	Szkudlarz P, POZ
21.	*Euerica*	*Brachycallis*	*E*. *cinerea* L.	Scotland, Cabrach, S Duffton	N 57°19'23.8" W 2°59'22.2	Szkudlarz P, POZ
22.	*Euerica*	*Eremocallis*	*E*. *tetralix* L.	Scotland, New Port, S Dundee	N 56°25’45” W 2°56’03,3”	Szkudlarz P, POZ
23.	*Chlamydanthe*	*Geissostegia*	*E*. *imbricata* L.	RSA, Boskloof	S 34°24’30” E 19°40’22”	SWO 119, POZG
24.	*Chlamydanthe*	*Elytrostegia*	*E*. *lasciva* Salisb.	RSA, Boskloof	S 34°24’30” E 19°40’22”	SWO 117, POZG
25.	*Chlamydanthe*	*Elytrostegia*	*E*. *diosmifolia* Salisb.	RSA, Table Montain, Castle Rock, 760 m a.s.l.	S 33°59'1,256" E 18°25'0,878"	SWO 17, POZG
26.	*Chlamydanthe*	*Eurystegia*	*E*. *monsoniana* L.f.	RSA, Jonaskop, 1000 m a.s.l.	S 33°56’28” E 19°31’14”	SWO 189, POZG
27.	*Chlamydanthe*	*Eurystegia*	*E*. *lanipes* Guthrie&Bolus	RSA, Pilaarkop,	S 34°05’11” E 19°51’10”	SWO 155, POZG
28.	*Chlamydanthe*	*Adelopetalum*	*E*. *nabea* Guthrie & Bolus	RSA, Prince Alfred’s Pass	S 33°45’30” E 23°09’45”	SWO 207, POZG
29.	*Chlamydanthe*	*Trigemma*	*E*. *plumigera* Bartl.	RSA, Koeëlbaai,	S 34°16’0” E 18°51’01”	SWO 96, POZG
30.	*Chlamydanthe*	*Trigemma*	*E*. *baccans* L.	RSA, Kirstenbosch, slope of the Table Mountain Mo	S 33°58’54” E 18°25’57,8”	SWO 14, POZG
31.	*Platystoma*	*Polycodon*	*E*. *sparsa* Lodd.	RSA, Prince Alfred’s Pass	S 33°45’30” E 23°09’45”	SWO 207A, POZG
32.	*Platystoma*	*Eurystoma*	*E*. *lucida* Salisb.	RSA, Elandskloof, Wolseley,	S 33°58’54” E 18°08’17”	SWO 31, POZG
33.	*Platystoma*	*Eurystoma*	*E*. *adnate* L.Bolus	RSA, head of Bavians Kloof on Jonascop	S 33°57’36” E 19°31’07”	SWO 182, POZG
34.	*Platystoma*	*Gamochlamys*	*E*. *passerinae* Montin	RSA, Uniondale Pourt,	S 33°42’10” E 23°09’30”	SWO 210, POZG
35.	*Platystoma*	*Gamochlamys*	*E*. *melanthera* L.	RSA, Langeberg Tradoupas, 351 m a.s.l.	S 33°58’35” E 20°42’09”	SWO 194, POZG
36.	Minor Genera		*E*. *glabella* Thunb.	RSA, 15 km S of Cape Town, Steenberg, Silverberg	S 34°05’28” E 18°25’14,4”	SWO 1, POZG
37.	Minor Genera		*E*. *globiceps* (N.E.Br.) EGH.Oliv	RSA, Groot Hagelkraal	S 34°40’56” E 19°34’29”	SWO 133, POGZ
38.	Minor Genera		*E*. *axillaris* Thunb.	RSA, 15 km S of Cape Town, Steenberg, Silverberg	S 34°05’47,5” E 18°25’30,6”	SWO 4, POZG
39.	Minor Genera		*E*. *plumosa* Thunb.	RSA, Mamre, 173 m a.s.l.	S 33°31’11” E 18°27’55”	SWO 39, POZG
40.	Minor Genera		*E*. *totta* Thunb.	RSA, Theevaterklof	S 33°58’09,2” E19°10’01,5”	SWO 49, POZG
41.	Minor Genera		*E*. *puberuliflora* E.G.H. Oliv.	RSA, Boskloof	S 34°24’30” E 19°40’22”	SWO 112, POZG
42.	Madagaskar		*E*. *parkeri* (Baker) Dorr&E.G.H.Oliv.	Madagascar, Antsirabe	S 19°53’24” E 47°2’27,82”	Szkudlarz P, POZ
43.	Madagaskar		*E*. *goudotiana* (Klotzsch) Dorr&E.G.H.Oliv Dorr&E.G.H.Oliv.	Madagascar, Antsirabe	S 19°53’24” E 47°2’27,82”	Szkudlarz P, POZ
44.	Madagaskar		*E*. *cryptoclada* (Baker)Dorr&E.G.H.Oliv.	Madagascar, Ambohibary	S 19°37’9,12” E 47°6’36,72”	Szkudlarz P, POZ
45.	Madagaskar		*E*. *baroniana* Dorr&E.G.H.Oliv.	Madagascar, Ambohibary	S 19°37’9,12” E 47°6’36,72”	Szkudlarz P, POZ

Herbarium of Department of Plant Taxonomy (POZ); Herbarium of Natural History Collections (POZG) in Adam Mickiewicz University in Poznań. SWO—**S**zkudlarz P., **W**iland-Szymańska J., **O**liver EGH—the persons who have collected plant material in RSA in **20**05.

## Material and methods

### Palynological analysis

They were analysed 45 *Erica* species; 39 species with terads and six species with monads. A list of the analysed taxa, with their affiliation to particular subgenera and sections, is shown in Table 1.

In this paper, the taxonomic classification of the studied taxa from *Erica* genus was adopted from publication [10], with further changes [11, 12, 21, 40]. The sections names for species from South Africa follows [9] and for species fom Europe follows [41]. The verification of the taxa from South Africa was made by Prof. Edward Oliver (Stellenbosch Herbarium, National Botanical Institute, South Africa), the taxa from Europe and Madagascar were reviewed by Prof. Piotr Szkudlarz (Adam Mickiewicz University in Poznań, Poland)—the outstanding taxonomists specializing in the *Erica* genus. In Table 1, heather species from Minor Genera, which were included in the *Erica* genus [6], were excluded from the system because, so far, they had not been allocated to any section. Similar approach was adopted in the case of species derived from Madagascar, which were earlier placed in the *Philippia* genus [21].

The plant material were collected by from 45 natural *Erica* sites in years: 2005 (South Africa—Republic of South Africa), 2010 (Spain) and 2014 (Madagascar) (Table 1).

Several randomly selected inforesecences or flowers were collected from each individual plant. For each analysed species 30 mature, correctly formed pollen grains was measured by LM. In total, 1350 pollen grains were analysed.

All samples were acetolysed according to Erdtman’s method [42], with insignificant modifications. Shrivelled, ground flowers or inflorescences were blent with acetolysis mixture, consisted of acetic anhydrite (9 portions) and concentrated sulphuric acid (1 portion). Then suspension was heated to boiling point and kept in water bath for 2–3 minutes. Next the samples were centrifuged in acetolysis mixture, washed with acetic acid and centrifuged again. Finally they were both mixed with alcohol 96% and centrifuged four times. Prepared material was divided into two parts. One half was immersed in alcohol solution of glycerine (for LM) and the other in ethyl alcohol 96% (for SEM).

The observations were carried out with light microscope (Biolar 2308) and scanning electron microscope (Philips SEM-515) on acetolysed pollen grains and the measurements were carried out with LM on acetolysed pollen grains to. Most of the measurements were done on the LM at magnification of x640. Pollen grains were prepared in glycerine jelly and measured using the eyepiece (ocular) with scale and then measurement results were recalculated into micrometers by multiplying them by 2.

The pollen grains were analysed for 11 quantitative features, i.e. tetrad diameter (D), length of polar axis (P) and equatorial diameter (d in tetrads or E in monads), length of ectoaperture (2f in tetrads or Le in monads), apocolpial (Ex) and septal (Se) exine thickness and for a monads P/E, Le/P, Ex/E ratios and for a tetrads D/d, P/d, 2f/D, Ex/Se and Ex/d ratios; and the following qualitative ones: outline, shape and exine ornamentation. Tetrads were measured in the distal face and monads in polar view.

The palynological terminology follows [37, 43].

### Statistical analysis

Monads and tetrads were analysed independently. Firstly, the normality of the distributions of the studied traits (for monad: P, E, Le, Ex, P/E, Le/P and Ex/E; for tetrad: D, d = E, P, 2f = Le, Se, Ex, D/d, P/d, 2f/D and Ex/Se) were tested using Shapiro-Wilk’s normality test [44]. Multivariate analysis of variance (MANOVA) was performed on the basis of following model using a procedure MANOVA in GenStat 17th edition: Y = XT+E, where: Y is (*n*×*p*)–dimensional matrix of observations, *n* is number of all observations, *p* is number of traits (in this study *p* = 7 for monad and *p* = 11 for tetrad), X is (*n*×*k*)–dimensional matrix of design, *k* is number of species (in this study *k* = 6 and *k* = 39 for monad and tetrad, respectively), T is (*k*×*p*)–dimensional matrix of unknown effects, E–is (*n*×*p*)–dimensional matrix of residuals. Nextly, one-way analyses of variance (ANOVA) were performed in order to verify the zero hypothesis on a lack of species effect in terms of values of observed traits, for each trait independent, on the basis of the following model: *y*_*ij*_ = *μ*+*τ*_*i*_+*ε*_*ij*_, where: *y*_*ij*_ is *j*th observation of *i*th species, *μ* is grand mean, *τ*_*i*_ is effect of *i*th species and *ε*_*ij*_ is an error observation. The minimal and maximal values of traits as well as arithmetical means and coefficients of variation–CV (in %)–were calculated. Moreover, the Fisher’s least significant differences (LSDs) were also estimated at the significance level α = 0.001. The relationships between observed traits were assessed on the basis of Pearson’s correlation coefficients using FCORRELATION procedure in GenStat 17th edition. Results were also analysed using multivariate methods. The canonical variate analysis was applied in order to present multitrait assessment of similarity of tested species in a lower number of dimensions with the least possible loss of information [45]. This makes it possible to illustrate variation in species in terms of all observed traits in the graphic form. Mahalanobis’ distance was suggested as a measure of “polytrait” species similarity [46], whose significance was verified by means of critical value D_α_ called “the least significant distance” [47]. Mahalanobis’ distances were calculated for species. The differences among analysed species were verified by the cluster analysis using the nearest neighbour method and Euclidean distances [48]. Difference between values of P/E for monads and P/d for tetrads was tested on the based of *t*-test. All the analyses were conducted using the GenStat v. 17 statistical software package [49].

## Results

The study was conducted on 45 *Erica* species which represent alle five subgenera and 22 sections of the genus *Erica* (Table 1). The pollen grains of *Erica* studied species were usually dispersed as tetrahedral tetrads (39 species), rarely as monads (6 species).

**Fig 1:**
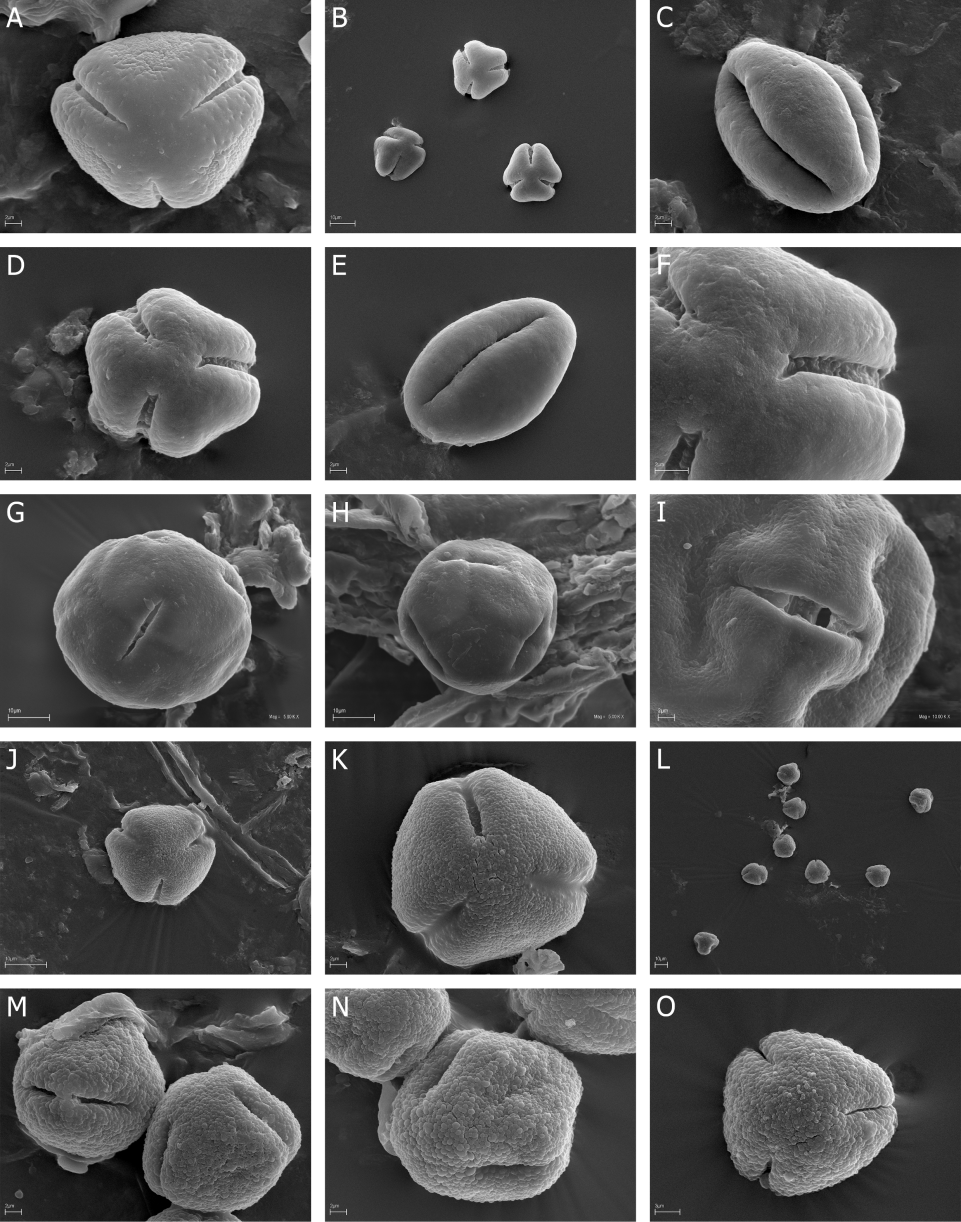
A-O. *E*. *fastigiata*, A. A, monad (pollen grain) in polar view, psilate at the poles and along the colpi; *E*. *glabella*, B-C. B, three pollen in polar view; C, equatorial view with two colpi; *E*. *globiceps*, D-F. D, polar view with three colpi; E, equatorial view; F, polar area with three colpi; *E*. *nabea*, G-I. G, polar view with three short colpi, H—equatorial view, I—colpus with opened porus; *E*. *plumose*, J-K. J, polar view with three colpi, K, granulate-fossulate exine ornamentation; *E*. *puberuliflora* L-O. L, pollen grains in polar and equatorial view, M, two pollen grains in polar and equatorial view, N, granulate-fossulate exine ornamentation O, granulate-fossulate exine ornamentation.

**Fig 2:**
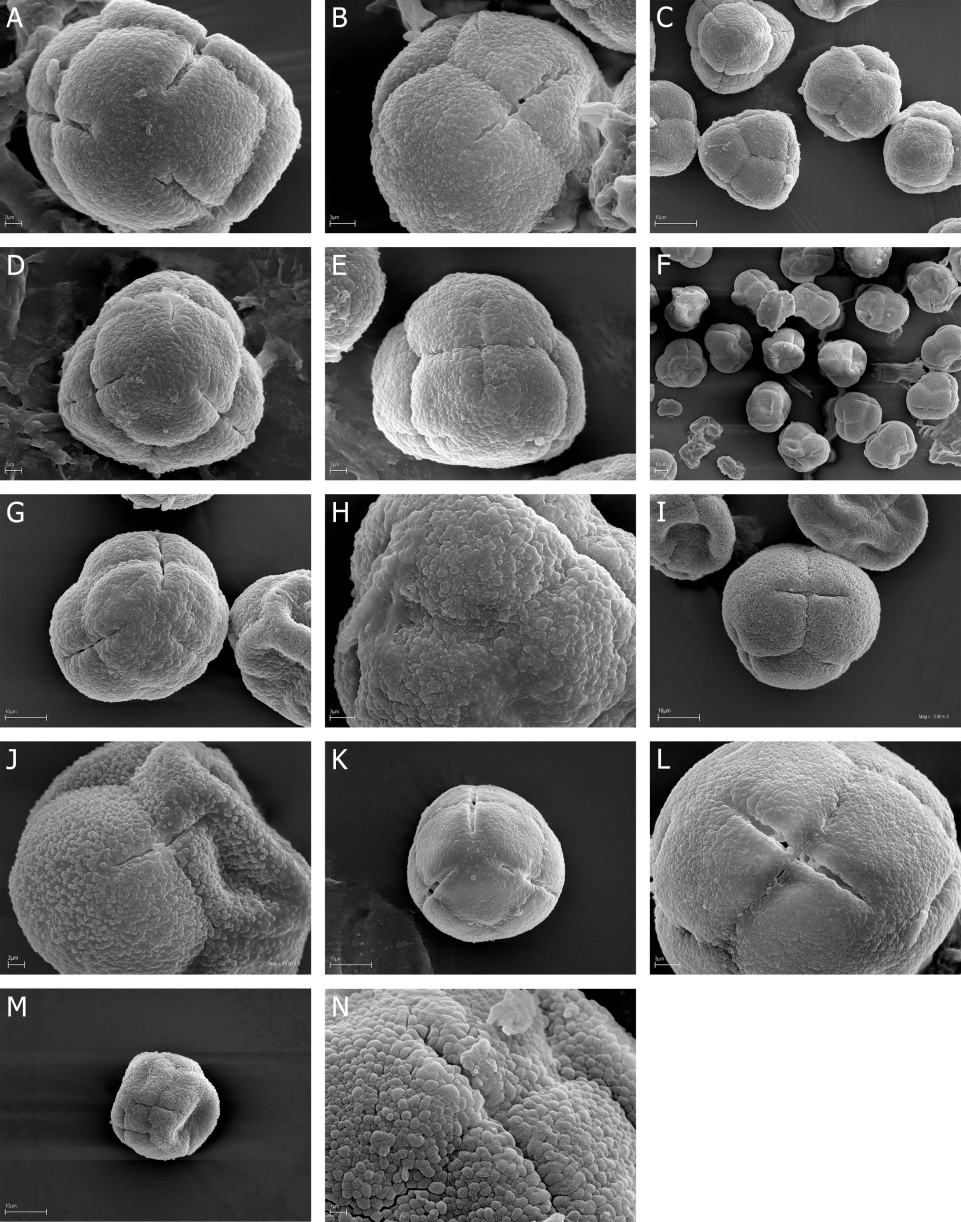
A-N. *E*. *adnata*, A-B. A, tetrad in proximal face with three colpori, B, tetrad in distal face with one colpori; *E*. *arborea*, C-E. C, A few tertads in proximal and distal face, D, tetrad in proximal face with colpori, E, tertad in lateral view; *E*. *australis*, F-H. F, over a dozen of tertads in proximal and distal face, G, tetrad in proximal face with three, narrow colpori, H, granulate-fossulate exine ornamentation with clearly visible, numerous microgranules; *E*. *axillaris*, I-J. I, tertad in lateral view with colporus, J, granulate exine ornamentation without microgranules; *E*. *baccans*, K-L. K, tetrad in proximal face with three, opened colpori, L, tertad in lateral view with colporus; *E*. *baroniana*, M-N. M, tertad in lateral view with closed colpori, N, granulate-fossulate exine ornamentation with clearly visible, numerous microgranules.

**Fig 3:**
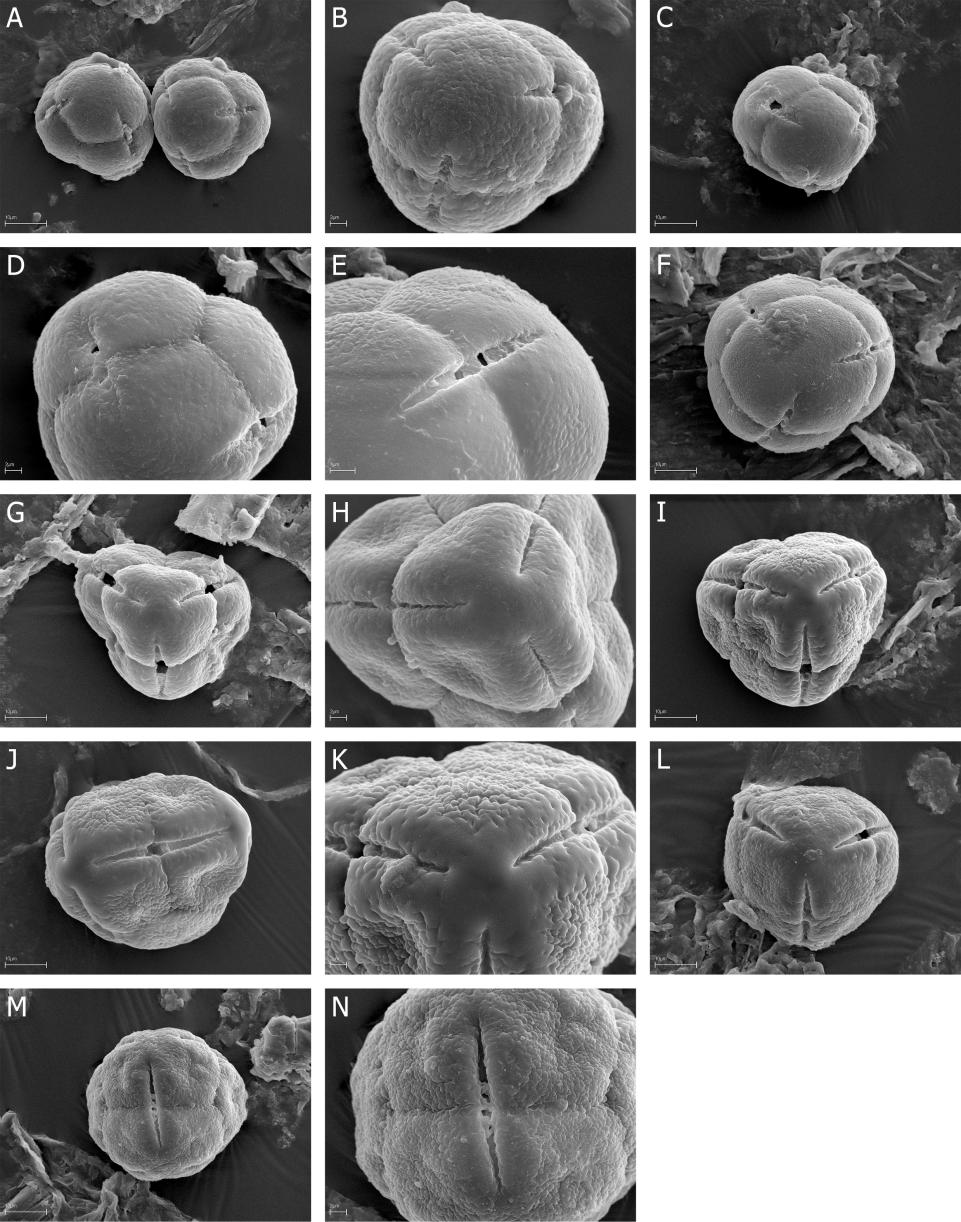
A-N. *E*. *caffra*, A-D. A, two tetrads in proximal face with colpori, B, tetrad in proximal face with three, closed colpori, C, opened colpori, D, two opened colpori and psilate exine ornamentation with microgranules; *E*. *cinerea*, E-F. E, tetrad in proximal face with three, opened colpori, F, opened colpori of two neighboring pollen grains; *E*. *coccinea*, G-H. G, tetrad in proximal face with three, opened colpori, H—tetrad in proximal face with closed, narrow colpori; *E*. *curviflora*, I-K. I, tetrad in proximal face, granulate-fossulate exine ornamentation with psilate “belts” at the poles and along the colpi, J, tetrad in lateral view with narrow colporus, K, proximal pole with three narrow opened colpori; *E*. *denticulate*, L-N. L, tetrad in proximal face with three narrow, opened colpori, M, tetrad in lateral view with colporus, N, narrow colporus.

**Fig 4:**
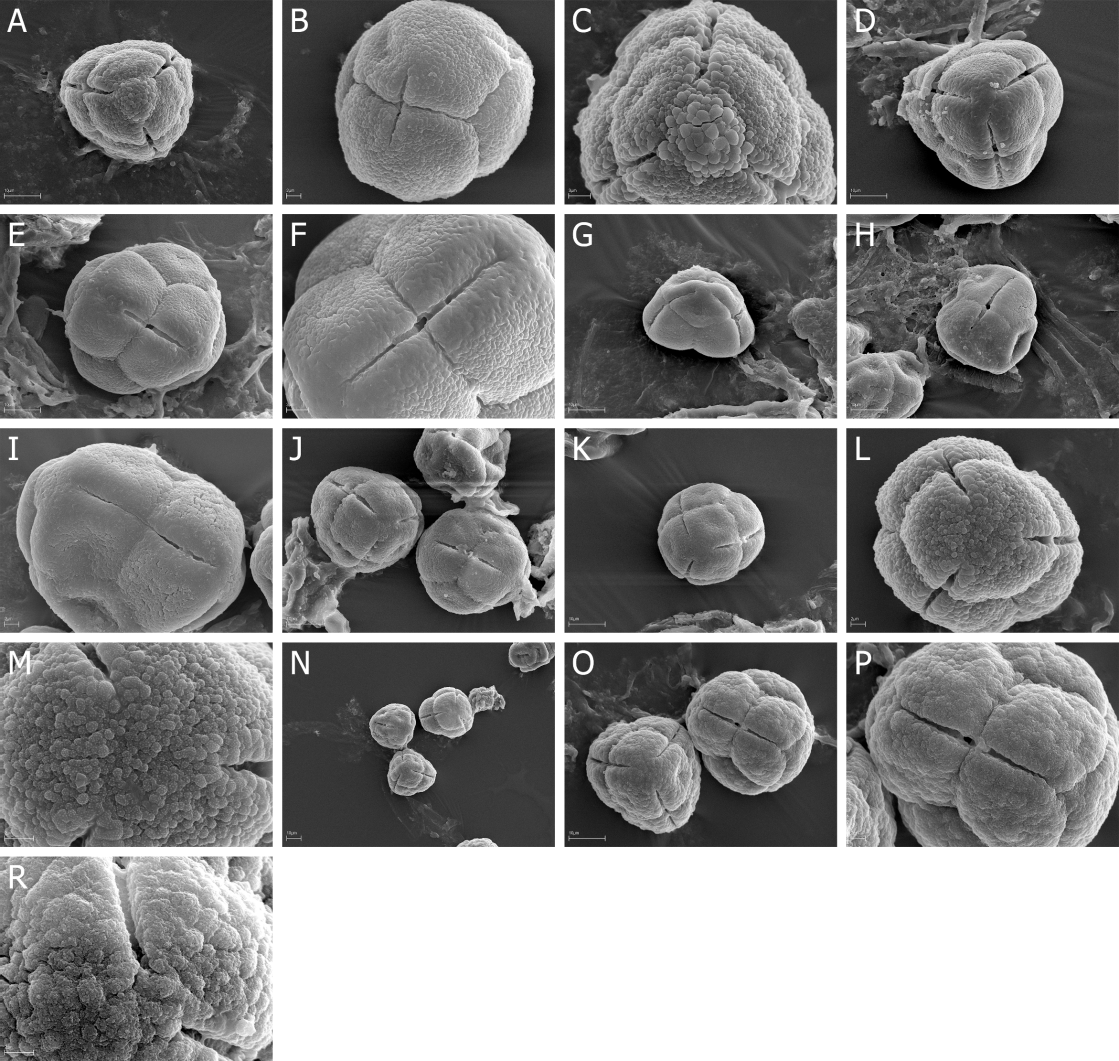
A-R. *E*. *diosmifolia*, A-C. A, tetrad in proximal with narrow colpori of neighboring pollen grains, B, tetrad in lateral view with narrow colporus, C, proximal pole with fossulate exine ornamentation; *E*. *discolor*, D-F. D, tetrad in proximal face, exine with psilate “belts” at the poles and along the colpi, E, tetrad in lateral view with narrow, opened colporus, F, narrow, opened colporus and exine ornamentation; *E*. *erigena* G-I. G, tetrad in proximal face, H, tetrad in lateral view with narrow, opened colporus, I, narrow, closed colporus; *E*. *hirtiflora*, J-K. J, two tetrads in proximal face and lateral view, K, tetrad in proximal face with narrow colpori; *E*. *hispidula*, L-M. L, tetrad in proximal face, granulate-fossulate exine ornamentation, M, granulae and fossulae with very numerous microgranules; *E*. *imbricate*, N-R. N, three tetrads in proximal and distal face, O, two tetrads in proximal face and lateral view, P, narrow, closed colporus, R, proximal pole, granulate-fossulate exine ornamentation with very numerous microgranules.

**Fig 5:**
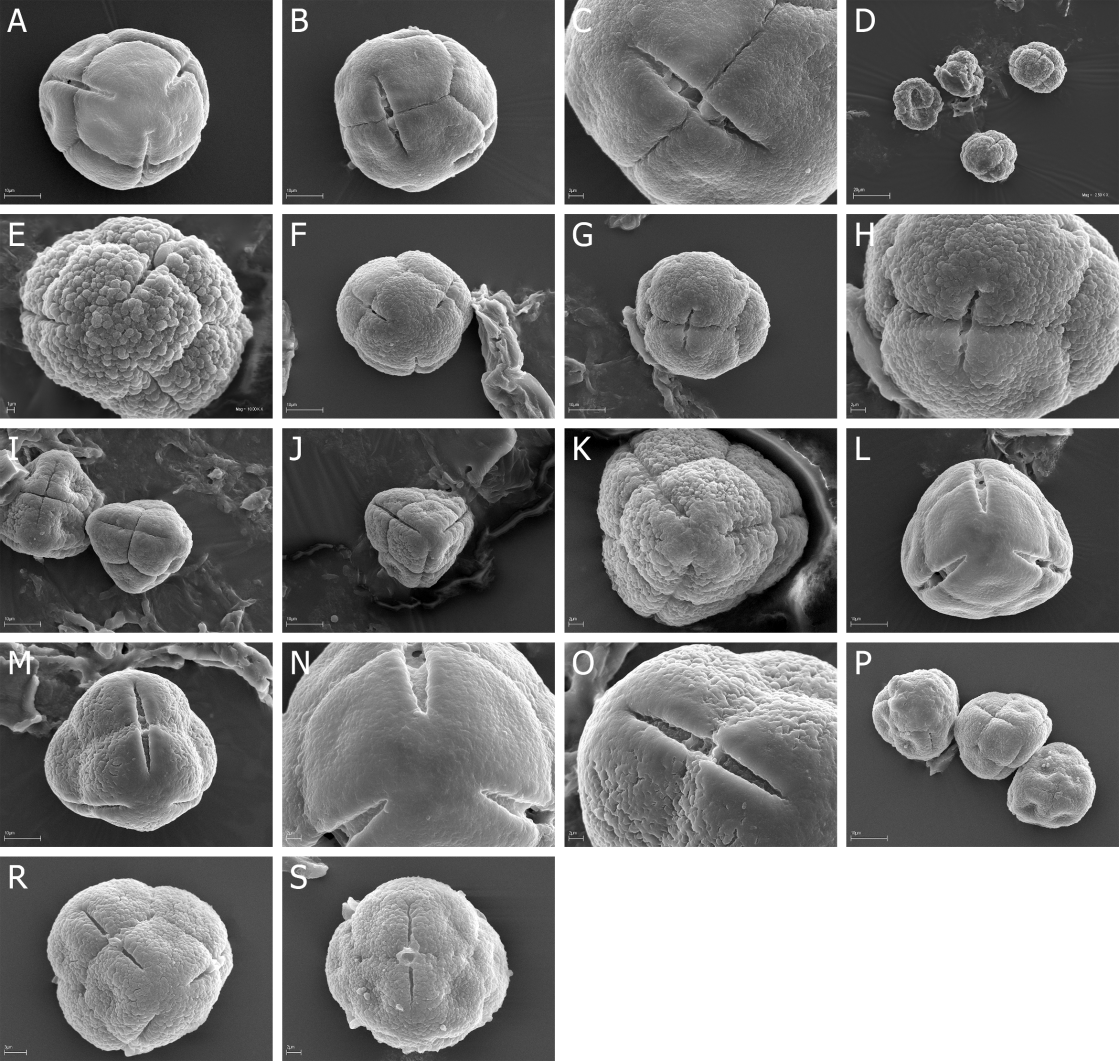
A-S. *E*. *lanipes*, A-C A, tetrad in proximal face with psilate exine ornamentation, B, tetrad in lateral view, C, narrow, opened colporus; *E*. *lasciva*, D-E D, four tetras in proximal and distal face, E, fossulate exine ornamentation with very numerous microgranules; *E*. *lucida* F-H. F, tetrad in proximal face with narrow colpori, G, tetrad in lateral view, H, narrow, opened colporus; *E*. *melanthera*, I-K. I, two tetrads in lateral view, J, tetrad in proximal face, K, proximal pole with narrow closed colpori; *E*. *monsoniana*, L-N. L, tetrad in proximal face with narrow, opened colpori, M, tetrad in proximal face with narrow, closed colpori, N, proximal pole with microgranules, O, exine with psilate “belts” along the colporus; *E*. *parviflora*, P-S. P, two tetrads in proximal and distal face, R, tetrad in proximal face with narrow, closed colpori, S, tetrad in lateral view.

**Fig 6:**
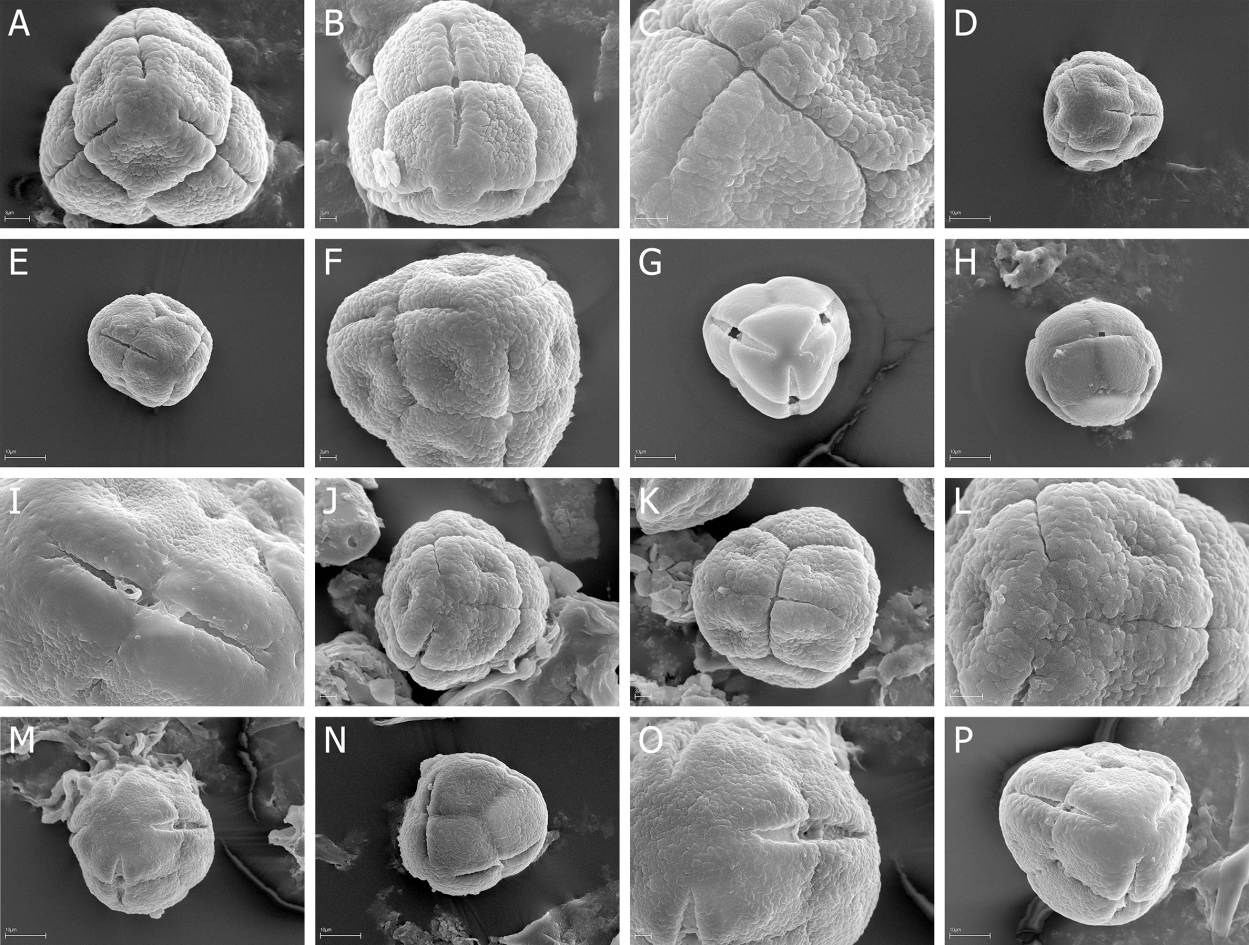
A-P. *E*. *passerinae*, A-C. A, tetrad in proximal face; granulate-fossulate exine ornamentation with psilate “belts” at the poles and along the colpi, B, tetrad in lateral view, C, granulate-fossulate exine ornamentation with very numerous microgranules; *E*. *peziza*, D-F. D, tetrad in proximal face, E, tetrad in lateral view, F, granulate-fossulate exine ornamentation; *E*. *plukenetii*, G-H. G, tetrad in proximal face with opened colpori and psilate exine ornamentation, H, tetrad in lateral view with opened colpore; *E*. *regia*, I-J. I, tetrad in proximal face; granulate-fossulate exine ornamentation, psilate at the poles and along the colpi, J, colporus; *E*. *sparsa*, K-M. K, tetrad in proximal face, L, tetrad in lateral view with closed colporus, M, fossulae with very numerous microgranules; *E*. *tenella*, N-P. N, tetrad in proximal face, O, tetrad in distal face, P, granulate-fossulate exine ornamentation and colporus.

**Fig 7:**
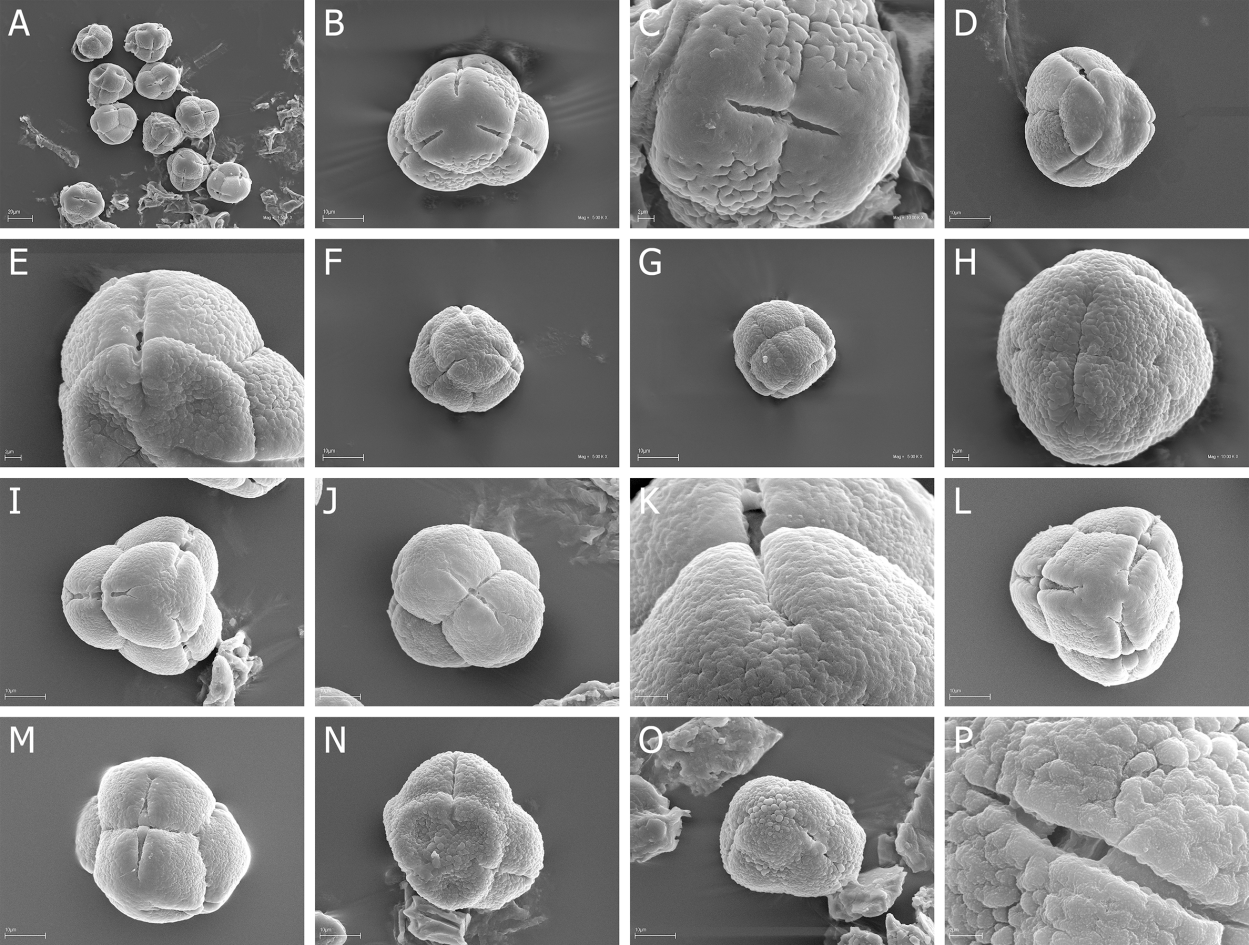
A-P. *E*. *tetralix*, A-C. A, tetrads in proximal and distal faces and in lateral view, B, tetrad in proximal face; exine ornamentation granulate-fossulate and psilate at the poles and along the colpi, C, colporus and very numerous microgranules; *E*. *totta*, D-E. D, tetrad in proximal face; exine ornamentation granulate-fossulate and psilate at the poles and along the colpi, E, opened colporus; *E*. *trichophylla*, F-H F, tetrad in proximal face, G, tetrad in lateral view, H, closed colporus; *E*. *umebellata*, I-K. I, tetrad in proximal face, J, tetrad in lateral view, K, granulate-fossulate exine ornamentation with very numerous microgranules; *E*. *vestita*, L-N. L, tetrad in proximal face; exine ornamentation granulate-fossulate and psilate at the poles and along the colpi, M, tetrad in lateral view, colporus with a bridge; *E*. *zwartbergensis*, N-P. N, tetrad in proximal face, O, tetrad with numerous fossulae, P, fossulae with very numerous microgranules.

Quantitative features of pollen grains are summarized in Table 2 (monads) and in Tables 3–5 (tetrads) and illustrated with scanning electron micrographs (Fig 1 - monads, Figs 2–7 - tetrads).

**Table 2:** Minimal, maximal and mean values as well as coefficients of variation (CV) for observed traits of species with monads.

Species	P			E			Le			Ex		
	min-max	Mean	CV	min-max	Mean	CV	min-max	Mean	CV	min-max	Mean	CV
*E*. *fastigiata*	24–30	27.80 a	6.64	20–28	23.93 a	7.44	18–26	21.20 a	10.97	1–2	1.833 a	20.68
*E*. *glabella*	20–30	25.73 bc	9.07	16–24	20.27 b	7.66	14–20	17.13 c	9.04	1–3	1.633 a	34.05
*E*. *globiceps*	20–30	24.93 c	8.87	16–22	18.60 c	8.54	14–22	18.80 bc	11.03	1–2	1.833 a	20.68
*E*. *nabea*	20–26	21.67 d	7.31	14–18	16.53 d	8.95	16–22	18.33 bc	9.54	1–2	1.133 b	30.51
*E*. *plumosa*	24–30	27.40 ab	5.12	20–28	23.20 a	7.71	16–24	19.87 ab	9.51	1–2	1.901 a	16.06
*E*. *puberuliflora*	20–30	24.20 a	8.78	16–24	20.80 b	6.96	16–24	18.27 bc	11.03	1–2	1.667 a	28.76
LSD_0.001_		1.68			1.39			1.69			0.36	
*F* statistic		40.22***			89.04***			15.90***			13.70***	
Species	P/E			Le/P			Ex/E					
	min-max	Mean	CV	min-max	Mean	CV	min-max	Mean	CV			
*E*. *fastigiata*	1–1.36	1.165 c	6.82	0.64–0.87	0.763 b	8.82	0.038–0.1	0.077 b	22.11			
*E*. *glabella*	1–1.5	1.273 ab	8.90	0.57–0.82	0.669 c	9.79	0.045–0.136	0.081 b	33.10			
*E*. *globiceps*	1.11–1.63	1.345 a	8.86	0.53–0.90	0.759 b	12.74	0.05–0.125	0.099 a	22.33			
*E*. *nabea*	1.11–1.57	1.318 a	9.08	0.67–1	0.848 a	8.52	0.056–0.125	0.069 b	28.07			
*E*. *plumosa*	0.94–1.4	1.186 bc	7.65	0.6–0.8	0.725 bc	8.15	0.042–0.1	0.083 ab	18.49			
*E*. *puberuliflora*	1–1.5	1.167 c	9.40	0.67–0.9	0.756 b	8.86	0.045–0.1	0.080 b	27.31			
LSD_0.001_		0.092			0.062			0.018				
*F* statistic		16.98***			19.47***			7.04***				

P—length of polar axis; E—equatorial diameter; Le—length of ectocolpi; Ex—thickness of exine.

**Table 3:** Minimal, maximal and mean values as well as coefficients of variation (CV) for observed traits of species with tetrads.

Species	D			d = E			P			2f = Le		
	min-max	Mean	CV	min-max	Mean	CV	min-max	Mean	CV	min-max	Mean	CV
*E*. *adnata*	30–36	33m	5.90	20–28	24.8ghij	8.36	10–18	14.67op	19.06	14–22	16.87klm	13.09
*E*. *arborea*	24–30	27.33rs	5.20	18–22	19.93p	4.92	14–20	16.07lmno	10.58	12–18	14.87nop	10.99
*E*. *australis*	40–50	47.13e	5.31	30–34	32.13bc	3.98	16–26	20.2hij	13.09	20–30	25.13d	8.28
*E*. *axillaris*	30–44	36.67kl	8.15	22–30	26efgh	6.70	16–24	19.53ij	10.31	14–20	16.87klm	11.52
*E*. *baccans*	36–44	40.4h	3.99	22–32	26.47efg	9.66	18–30	21.8efgh	11.13	16–22	18.73hij	9.08
*E*. *baroniana*	26–32	28.8pqr	5.35	18–24	20.67nop	7.33	10–18	14.67op	11.51	16–22	18.47hijk	10.13
*E*. *caffra*	28–36	31no	8.26	20–28	22.87klm	6.77	14–20	16.8klmn	8.62	10–18	13.4pqr	16.23
*E*. *cinerea*	42–54	49.13bcd	6.11	28–44	36.4a	9.83	22–34	25.73bc	11.50	24–30	27.2c	8.10
*E*. *coccinea*	20–48	40.93gh	11.24	24–30	27.07def	6.36	18–26	22.67def	8.46	16–24	19.47fgh	10.07
*E*. *cryptoclada*	36–44	39.13hij	4.78	24–32	28.47d	7.97	16–26	21.73efgh	11.52	18–26	21.6e	9.23
*E*. *curviflora*	46–50	48.13de	2.66	30–36	33.07b	5.20	18–28	24.4cd	8.17	26–32	30.07b	5.92
*E*. *denticulata*	40–46	42.55fg	5.31	26–34	30.83c	5.35	20–30	24.41cd	9.64	18–24	21.59e	8.35
*E*. *diosmifolia*	30–34	31.47mno	4.70	18–26	21.27mnop	10.02	14–22	17.53kl	14.27	14–20	17.07jklm	9.60
*E*. *discolor*	42–56	49.73bc	6.98	28–36	32.93b	7.77	20–30	25.67bc	9.17	26–32	28.87b	5.95
*E*. *erigena*	28–34	31.73mn	4.89	16–22	20.47op	7.56	10–18	12.33q	17.08	16–24	19.53fgh	9.95
*E*. *goudotiana*	34–44	37.93ijk	5.27	22–32	26.67ef	8.43	18–28	21.13fghi	11.84	18–24	20.73ef	10.89
*E*. *hirtiflora*	26–34	31.6mn	6.31	20–28	23.33jkl	8.52	14–20	16.8klmn	8.62	12–20	16.33lmn	11.64
*E*. *hispidula*	28–34	30.53nop	5.14	20–24	21.6mnop	5.10	12–22	16.73klmn	11.53	12–18	15.53mno	11.06
*E*. *imbricate*	34–38	36l	3.86	24–28	26.13efgh	3.99	14–24	20.2hij	11.73	14–22	18.53hijk	9.79
*E*. *lasciva*	26–36	31.2mno	8.18	18–28	21.27mnop	10.90	10–22	15.6mno	16.63	12–18	14.93nop	12.05
*E*. *lucida*	36–42	39.13hij	4.97	16–30	24ijk	13.13	16–28	20.47ghij	13.00	16–26	19.13fghi	11.87
*E*. *melanthera*	30–34	31.47mno	3.31	20–24	21.8lmno	4.41	14–20	17klmn	8.60	16–22	18.27hijk	10.65
*E*. *monsoniana*	46–58	50.47b	5.17	32–42	36.27a	6.27	18–30	26.4b	7.81	26–34	29.8b	6.68
*E*. *parkeri*	28–34	31no	5.29	18–26	22.07lmno	9.66	10–20	15.47no	16.96	10–18	12.13r	19.33
*E*. *parviflora*	24–30	28.07qr	5.12	18–22	20.07p	6.13	12–20	15.87lmno	10.95	10–18	13qr	16.54
*E*. *passerinae*	26–32	29.6opq	5.44	20–24	20.8nop	5.98	12–18	15.53mno	10.52	16–22	18.33hijk	9.54
*E*. *peziza*	28–34	30.53nop	5.42	20–26	22.07lmno	8.41	14–18	16.2lmno	6.76	16–20	18.13hijk	7.63
*E*. *plukenetii*	34–42	37.07kl	5.62	20–28	24.93ghij	7.52	18–26	20.93fghi	9.95	18–24	20.47efg	6.63
*E*. *plumigera*	34–40	37.07kl	5.06	20–30	24.67hij	10.06	18–26	22.2efg	9.86	16–22	18.87ghi	7.20
*E*. *regia*	42–54	48.41cde	4.60	30–36	33.24b	5.43	20–30	25.93bc	9.10	24–32	28.9b	6.02
*E*. *sparsa*	24–28	26.13s	4.89	16–20	18q	7.72	10–16	13.07pq	13.17	10–18	14.4opq	15.65
*E*. *tenella*	36–44	39.8hi	5.34	22–30	27.47de	6.88	20–28	22.4ef	9.20	18–24	19.67fgh	7.59
*E*. *tetralix*	36–44	40.73gh	4.56	24–32	28.73d	6.46	20–26	22.13efg	7.48	16–24	19.6fgh	8.64
*E*. *totta*	30–40	37.6jkl	7.31	20–32	25.6fghi	9.71	12–24	17.4klm	23.21	18–24	21.4e	9.55
*E*. *trichphylla*	28–34	30.6nop	4.59	20–24	22.33klmn	7.09	12–20	16.2lmno	9.91	16–20	17.8ijkl	8.00
*E*. *umbellata*	40–50	43.93f	5.79	24–30	28.47d	6.30	18–28	23.27de	9.70	16–22	18.53hijk	9.37
*E*. *vestita*	48–64	55.07a	5.29	30–42	36.4a	6.83	24–34	29.2a	7.54	28–42	33.87a	10.85
*E*. *viscidiflora*	32–44	40.07h	5.32	22–32	27.47de	7.39	14–22	18.6jk	14.72	16–22	18.8hi	8.65
*E*. *zwartbergensis*	32–44	36.93kl	7.21	22–30	25.93efgh	8.71	18–26	21.73efgh	9.28	16–20	18.8hi	7.18
LSD_0.001_		1.89			1.7			1.92			1.66	
*F* statistic		340.82***			185.63***			102.39***			202.56***	

D—tetrad diameter; d—equatorial diameter, P—length of polar axis; 2f = Le—length of ectoaperture; Ex—apocolpial exine thickness; Se—septal exine thickness.

* P<0.05

**P<0.01

***P<0.001.

**Table 4:** Minimal, maximal and mean values as well as coefficients of variation (CV) for observed traits of species with tetrads.

Species	Se			Ex			D/d			P/d		
	min-max	Mean	CV	min-max	Mean	CV	min-max	Mean	CV	min-max	Mean	CV
*E*. *adnata*	2–4	2.77cdefghij	29.53	1–2	1.17fghi	32.48	1.15–1.60	1.34i	8.00	0.38–0.80	0.59o	18.81
*E*. *arborea*	2–4	3.27bcd	25.34	1–2	1.17fghi	32.48	1.18–1.67	1.37ghi	6.24	0.70–1.00	0.81bcdefg	11.88
*E*. *australis*	2–6	3.13bcdef	33.25	1–2	1.53de	33.10	1.25–1.60	1.47bcdefgh	6.28	0.53–0.81	0.63mno	12.90
*E*. *axillaris*	2–4	2.6fghij	29.62	1–2	1.13fghi	30.51	1.15–1.69	1.41efghi	7.65	0.62–0.92	0.75defghijkl	10.21
*E*. *baccans*	2–4	3.1bcdef	21.35	2–2	2a	0.00	1.20–1.91	1.54bcd	10.68	0.67–1.15	0.83bcd	13.31
*E*. *baroniana*	2–4	2.43hij	25.73	1–2	1.7bcd	27.42	1.17–1.56	1.40fghi	6.48	0.50–0.82	0.71jkl	11.23
*E*. *caffra*	2–4	2.73cdefghij	25.30	1–2	1.1fghi	27.74	1.14–1.80	1.36hi	12.21	0.64–0.90	0.74ghijkl	8.25
*E*. *cinerea*	2–4	2.8cdefghij	34.33	1–2	1.13fghi	30.51	1.11–1.79	1.36hi	11.79	0.55–0.93	0.71ijkl	14.89
*E*. *coccinea*	2–4	2.63efghij	27.28	1–2	1.37ef	35.85	0.77–1.75	1.52bcde	11.70	0.64–1.00	0.84abc	7.94
*E*. *cryptoclada*	2–4	3.43b	22.54	1–2	1.1fghi	27.74	1.20–1.67	1.38fghi	7.81	0.57–0.92	0.77cdefghijk	10.60
*E*. *curviflora*	2–4	2.4ij	23.47	1–1	1i	0.00	1.33–1.67	1.46bcdefgh	5.02	0.56–0.88	0.74fghijkl	9.64
*E*. *denticulata*	2–4	2.83bcdefghij	29.99	1–2	1.21fghi	34.16	1.24–1.77	1.39fghi	8.88	0.67–1.07	0.79bcdefgh	12.00
*E*. *diosmifolia*	2–4	2.53fghij	22.55	2–2	2a	0.00	1.15–1.67	1.49bcdef	9.12	0.67–1.00	0.82bcde	9.94
*E*. *discolor*	2–4	2.7cdefghij	29.42	1–2	1.07ghi	23.78	1.17–1.80	1.52bcde	7.92	0.61–0.93	0.78cdefghij	8.36
*E*. *erigena*	2–4	2.83bcdefghij	30.86	1–2	1.07ghi	23.78	1.27–2.00	1.56ab	9.64	0.45–1.13	0.61no	23.69
*E*. *goudotiana*	2–4	3.27bcd	19.58	2–2	2a	0.00	1.13–1.83	1.43defghi	10.03	0.69–0.92	0.79bcdefghi	7.15
*E*. *hirtiflora*	2–4	2.77cdefghij	29.53	1–2	1.07ghi	23.78	1.18–1.60	1.36hi	8.94	0.57–0.90	0.72hijkl	11.36
*E*. *hispidula*	2–4	3.07bcdefg	25.59	1–2	1.07ghi	23.78	1.25–1.60	1.42efghi	5.43	0.55–1.00	0.78cdefghijk	12.93
*E*. *imbricate*	2–4	2.8cdefghij	23.73	1–2	1.03hi	17.68	1.29–1.58	1.38ghi	5.41	0.54–0.92	0.77cdefghijk	11.52
*E*. *lasciva*	2–4	3.07bcdefg	28.31	1–2	1.37ef	35.85	1.08–1.80	1.48bcdefg	11.23	0.56–1.00	0.74ghijkl	15.19
*E*. *lucida*	2–3	2.47ghij	20.57	1–2	1.87abc	18.52	1.20–2.50	1.66a	16.07	0.53–1.25	0.87ab	17.81
*E*. *melanthera*	2–4	2.47ghij	27.62	1–2	1.07ghi	23.78	1.33–1.60	1.45cdefghi	5.57	0.64–0.91	0.78cdefghij	9.59
*E*. *monsoniana*	2–4	2.37ij	25.98	1–2	1.17fghi	32.48	1.26–1.63	1.40fghi	6.14	0.53–0.81	0.73ghijkl	7.59
*E*. *parkeri*	2–4	2.7cdefghij	29.42	1–2	1.33efg	35.97	1.15–1.78	1.42efghi	9.02	0.50–0.90	0.70klm	13.46
*E*. *parviflora*	2–4	2.67defghij	31.65	1–2	1.03hi	17.68	1.18–1.56	1.40fghi	5.89	0.64–0.91	0.79bcdefghi	10.13
*E*. *passerinae*	2–4	2.3ij	23.26	1–2	1.07ghi	23.78	1.25–1.60	1.43efghi	6.57	0.60–0.90	0.75efghijkl	10.78
*E*. *peziza*	2–4	2.53fghij	22.55	1–2	1.17fghi	32.48	1.25–1.50	1.39fghi	4.90	0.58–0.82	0.74ghijkl	7.81
*E*. *plukenetii*	2–4	3.3bc	21.28	1–2	1.27efghi	35.50	1.36–1.80	1.49bcdef	7.30	0.69–1.00	0.84abc	8.46
*E*. *plumigera*	2–4	3.23bcde	23.94	2–2	2a	0.00	1.29–2.00	1.52bcde	9.45	0.67–1.30	0.91a	16.78
*E*. *regia*	2–4	3.07bcdefg	28.79	1–2	1.17fghi	32.80	1.33–1.69	1.46bcdefgh	6.36	0.59–0.93	0.78cdefghij	10.04
*E*. *sparsa*	2–3	2.27j	19.84	1–2	1.07ghi	23.78	1.30–1.63	1.46bcdefgh	7.07	0.56–0.89	0.73hijkl	11.76
*E*. *tenella*	2–4	2.9bcdefghi	20.94	2–2	2a	0.00	1.20–1.82	1.46bcdefgh	8.89	0.67–1.00	0.82bcdef	9.12
*E*. *tetralix*	2–4	2.8cdefghij	30.25	1–2	1.33efg	35.97	1.27–1.69	1.42efghi	7.70	0.67–1.00	0.77cdefghijk	8.65
*E*. *totta*	2–4	3.1bcdef	22.97	1–2	1.3efgh	35.85	1.15–1.90	1.48bcdefg	12.01	0.50–0.92	0.68lmn	18.46
*E*. *trichphylla*	2–4	2.6fghij	21.66	1–2	1.03hi	17.68	1.25–1.50	1.38ghi	6.09	0.58–0.91	0.731hijkl	10.00
*E*. *umbellata*	2–4	3.03bcdefgh	18.33	1–2	1.2fghi	33.90	1.40–1.83	1.55bc	7.21	0.64–0.93	0.82bcdef	9.26
*E*. *vestita*	4–6	4.17a	12.74	1–2	1.97ab	9.28	1.35–1.61	1.52bcde	4.35	0.68–0.94	0.80bcdefgh	7.55
*E*. *viscidiflora*	2–4	2.53fghij	22.55	1–2	1.67cd	28.76	1.25–1.75	1.47bcdefgh	7.66	0.47–0.79	0.68lmn	13.76
*E*. *zwartbergensis*	2–4	3.27bcd	19.58	2–2	2a	0.00	1.20–1.75	1.43defghi	10.73	0.73–1.08	0.84abc	9.72
LSD_0.001_		0.62			0.28			0.11			0.08	
*F* statistic		8.25***			35.21***			8.81***			16.79***	

See explanations to Table 3.

* P<0.05

**P<0.01

***P<0.001.

**Table 5:** Minimal, maximal and mean values as well as coefficients of variation (CV) for observed traits of species with tetrads.

Species	2f/D			Ex/Se			Ex/d		
	min-max	Mean	CV	min-max	Mean	CV	min-max	Mean	CV
*E*. *adnata*	0.39–0.69	0.51hijkl	12.37	0.25–1	0.46ghij	42.08	0.036–0.1	0.048jklmn	36.22
*E*. *arborea*	0.43–0.67	0.55efgh	12.41	0.25–1	0.38hij	43.70	0.045–0.111	0.059efghi	33.96
*E*. *australis*	0.40–0.68	0.543ghij	11.03	0.25–1	0.53defgh	41.95	0.029–0.067	0.048ijklmn	33.41
*E*. *axillaris*	0.32–0.67	0.46lmn	16.60	0.25–1	0.48ghij	43.98	0.033–0.083	0.0448jklmnop	30.13
*E*. *baccans*	0.38–0.61	0.46lmn	11.05	0.5–1	0.68abcd	24.11	0.063–0.091	0.076bc	9.80
*E*. *baroniana*	0.53–0.77	0.64a	10.82	0.25–1	0.74ab	35.93	0.045–0.111	0.083b	29.01
*E*. *caffra*	0.31–0.60	0.43mno	17.17	0.25–0.67	0.42ghij	27.35	0.036–0.1	0.049hijklmn	32.17
*E*. *cinerea*	0.48–0.67	0.55defgh	8.20	0.25–1	0.45ghij	42.24	0.023–0.056	0.031q	30.54
*E*. *coccinea*	0.36–0.90	0.48jkl	20.04	0.25–1	0.56cdefg	45.75	0.033–0.083	0.051fghijklm	37.29
*E*. *cryptoclada*	0.45–0.68	0.55defgh	10.77	0.25–0.67	0.34j	38.60	0.031–0.071	0.039nopq	26.93
*E*. *curviflora*	0.52–0.70	0.63ab	6.30	0.25–0.5	0.44ghij	19.88	0.028–0.033	0.030q	5.31
*E*. *denticulata*	0.39–0.60	0.51hijkl	10.38	0.25–1	0.45ghij	36.89	0.029–0.067	0.039mnopq	33.24
*E*. *diosmifolia*	0.44–0.67	0.54fghi	12.21	0.5–1	0.83a	21.47	0.077–0.111	0.095a	10.14
*E*. *discolor*	0.50–0.68	0.58bcdefg	7.43	0.25–0.67	0.42ghij	26.85	0.028–0.056	0.032pq	20.71
*E*. *erigena*	0.50–0.80	0.62abc	10.50	0.25–1	0.41ghij	38.00	0.045–0.1	0.052efghijk	25.78
*E*. *goudotiana*	0.45–0.67	0.55efgh	10.52	0.5–1	0.64bcdef	22.80	0.063–0.091	0.076bc	8.34
*E*. *hirtiflora*	0.38–0.64	0.52hijk	11.60	0.25–0.67	0.41ghij	28.14	0.036–0.083	0.046jklmno	21.82
*E*. *hispidula*	0.40–0.64	0.51hijkl	10.92	0.25–0.67	0.37ij	31.16	0.042–0.091	0.049ghijklmn	23.30
*E*. *imbricate*	0.39–0.61	0.52hijk	10.67	0.25–0.67	0.39hij	27.18	0.036–0.071	0.039lmnopq	15.69
*E*. *lasciva*	0.33–0.69	0.48klm	16.24	0.25–1	0.48ghij	45.69	0.036–0.1	0.065cde	35.83
*E*. *lucida*	0.38–0.68	0.49jkl	14.66	0.5–1	0.78ab	24.75	0.038–0.125	0.079b	23.40
*E*. *melanthera*	0.50–0.69	0.58bcdefg	11.14	0.25–1	0.46ghij	31.60	0.042–0.1	0.049hijklmn	26.03
*E*. *monsoniana*	0.52–0.68	0.59bcdef	5.95	0.25–1	0.51efghi	36.15	0.025–0.059	0.032pq	31.63
*E*. *parkeri*	0.29–0.60	0.39o	19.79	0.25–1	0.52efghi	38.78	0.038–0.1	0.062def	39.93
*E*. *parviflora*	0.33–0.67	0.47lmn	18.82	0.25–0.5	0.42ghij	25.73	0.045–0.091	0.052fghijk	15.54
*E*. *passerinae*	0.50–0.79	0.62abc	10.51	0.33–0.67	0.47ghij	16.29	0.042–0.1	0.052fghijk	26.04
*E*. *peziza*	0.47–0.67	0.60abcde	8.19	0.25–1	0.49fghij	45.47	0.038–0.1	0.054efghijk	35.68
*E*. *plukenetii*	0.48–0.719	0.55defgh	8.94	0.25–1	0.41ghij	46.53	0.036–0.091	0.051fghijkl	38.06
*E*. *plumigera*	0.40–0.61	0.51hijkl	8.65	0.5–1	0.66bcde	28.47	0.067–0.1	0.082b	10.44
*E*. *regia*	0.52–0.67	0.60abcd	6.06	0.25–1	0.42ghij	46.60	0.028–0.063	0.036opq	34.70
*E*. *sparsa*	0.36–0.69	0.55defgh	15.52	0.33–1	0.48ghij	26.17	0.050–0.125	0.060efgh	27.54
*E*. *tenella*	0.45–0.60	0.50ijkl	8.90	0.5–1	0.72ab	22.94	0.067–0.091	0.073bcd	7.35
*E*. *tetralix*	0.36–0.61	0.48klm	10.96	0.25–1	0.51efghi	44.68	0.031–0.077	0.046jklmno	35.74
*E*. *totta*	0.45–0.71	0.57cdefg	9.75	0.25–1	0.44ghij	45.75	0.031–0.091	0.052fghijk	39.02
*E*. *trichphylla*	0.47–0.67	0.58bcdefg	9.81	0.25–0.67	0.41ghij	23.92	0.042–0.091	0.047jklmno	19.35
*E*. *umbellata*	0.32–0.52	0.42no	10.99	0.25–1	0.42ghij	49.82	0.033–0.071	0.042klmnopq	31.29
*E*. *vestita*	0.52–0.75	0.62abc	9.29	0.25–0.5	0.48ghij	13.06	0.026–0.067	0.054efghij	11.98
*E*. *viscidiflora*	0.40–0.69	0.47klmn	12.55	0.25–1	0.70abc	37.99	0.031–0.091	0.061defg	31.36
*E*. *zwartbergensis*	0.40–0.59	0.51hijkl	10.74	0.5–1	0.64bcdef	22.80	0.067–0.091	0.078b	8.57
LSD_0.001_		0.05			0.15			0.012	
*F* statistic		28.59***			15.00***			37.23***	

See explanations to Table 3.

* P<0.05

**P<0.01

***P<0.001.

### Monads

Six (*E*. *fastigiata*, *E*. *glabella*, *E*. *globiceps*, *E*. *nabea*, *E*. *plumosa*, *E*. *puberuliflora*) of 45 studied *Erica* species occure isopolar monads (Fig 1A–1O).

All studied quantitative traits have a normal distribution as well as a multivariate normality. Results of MANOVA indicate that the species were significant (Wilk’s λ = 0.1044; F_35,709_ = 14.41; P < 0.0001) different for all seven traits. Results of analysis of variance for seven traits [P (F_5,174_ = 40.22), E (F_5,174_ = 89.04), Le (F_5,174_ = 15.90), Ex (F_5,174_ = 13.70), P/E (F_5,174_ = 16.98)], Le/P (F_5,174_ = 19.47)], Ex/E (F_5,174_ = 7.04)] at the significance level α = 0.001 (Table 2). Mean values and coefficients of variations (CV) for the observed traits indicate high variability among the species for which significant differences were found in terms of all analysed traits (Table 2).

The grains of this *Erica* species were 3 (4)—zonocolporate. The analysed pollen grains, according to Erdtman’s [22] pollen size classification, is medium (25.1–50 μm; 57,2%) or small (10–25 μm; 42,8%).

The average length of the polar axis (P) was 25.27 (**20**.00–30.00) μm. The smallest mean P were found for the pollen of *E*. *nabea* (21.67 μm), and the largest—for *E*. *plumosa* (27.4 μm) and *E*. *fastigiata* (27.8 μm) (Table 2).

The mean length of the equatorial diameter (E) was 20.52 (14.00–28.00). The shortest mean equatorial diameter occurred in the pollen of *E*. *nabea* (16.53 μm), while the longest was in *E*. *fastigiata* (23.93 μm; Table 2).

In all studied taxa, the outline in polar view was mostly circular, more rarely triangular or elliptic, whereas in equatorial view it was mostly elliptic.

**Fig 8:**
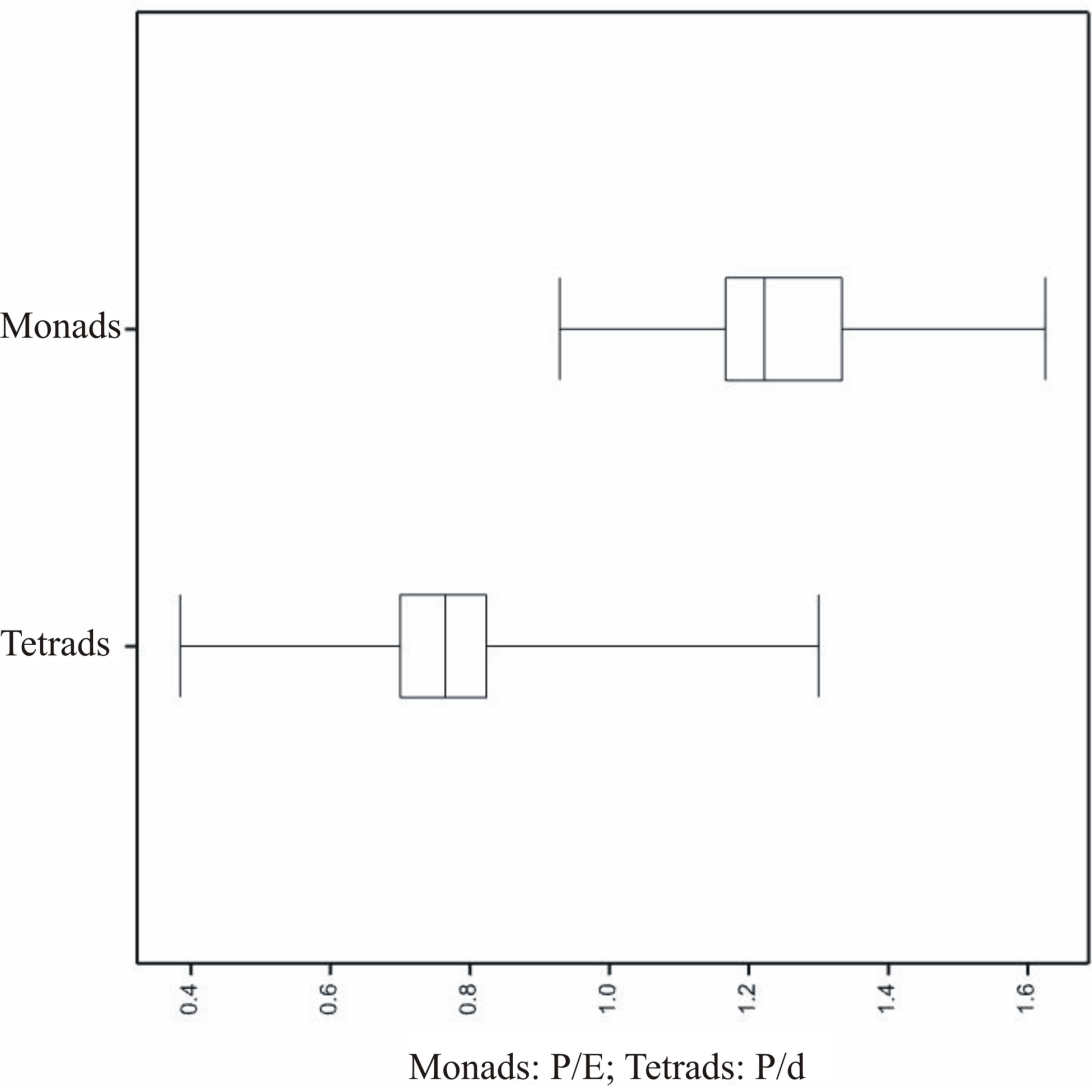
Box-and-whisker diagram of the values of diameter (D), classified by the tetrads’ species.

The most important difference between monads and tetrads were P/E and P/d ratios. In monads pollen grains were elongated and in tetrads flattened (Fig 8). In monads the mean P/E ratio was 1.24 (0.93–1.63) and ranged from 1.16 in *E*. *fastigiata* to 1.35 in *E*. *globiceps* (Fig 8). Pollen grains of the species examined were most frequently subprolate (60.6%), rarely prolate (20%) and prolate-spheroidal (16.1%) and very rarely spheroidal (2.8%) and oblate-spheroidal (0.6%).

The mean exine thickness (Ex) was 1,67 (1.0–3.0) μm (Table 2). Exine was thinnest in *E*. *nabea* (1.13 μm) and thickest (1.9 μm) in *E*. *plumosa*. Relative exine thickness (Ex/E) 0.08 (0.04–0.14), from 0.06 in *E*. *nabea* to 0.13 in *E*. *globiceps*.

Pollen grains usually possess three apertures—colpori. Ectoapertures—colpi were arranged meridionally, regularly, more or less evenly spaced, and were ussualy long; mean length 18.93 (14.00–26.00) μm (Table 2). On average, the length of colpi constituted 75% of the polar axis length. On average, the shortest colpi were found in *E*. *glabella* (17.13 μm), while the longest were found in *E*. *fastigiata* (21.2 μm). Colpi were fusiform in outline. Their width was variable and usually greatest in the equatorial region. Sculpturing of ectocolpus membranes varies from psilate to granulate. In *E*. *puberuliflora* the bridge is present. Endoapertures were usually located in the middle of colpi, less frequently asymmetrically, usually singly, very rarely in pairs. They were circular or elliptic in outline with irregular margins. Endoapertures usually distinct; opened or closed.

Exine ornamentation can be granulate and at the poles and along the colpori psilate (*E*. *fastigiata*), granulate-fossulate (*E*. *plumosa*, *E*. *puberuliflora*) or psilate with very numerous microgranules with the diameter usually 0.1 μm (*E*. *glabella*, *E*. *globiceps*) or with numerous microgranules with the diameter 0.1–0.3 μm (*E*. *nabea*).

On the basis of the pollen size and exine ornamentation all six studied *Erica* species with monads were distinguished (see: Pollen key).

The performed correlation analysis indicates statistically significant correlation coefficients of 13 out of 21 coefficients (Table 6). In the case of seven pairs of traits we observed positive correlation coefficients: P and E, P and Le, P and Ex, E and Le, E and Ex, Le and Le/P, P/E and Ex/E. This means that a value increase of one trait in a given pair leads to a value increase of the second trait. The negative correlation coefficients was observed between: P and Le/P, E and P/E, E and Le/P, Ex and P/E, Ex and Le/P, Le/P and Ex/E.

**Table 6:** Correlation matrix for the observed features of species with monads.

	P	E	Le	Ex	P/E	Le/P	Ex/E
P	1						
E	0.724***	1					
Le	0.502***	0.471***	1				
Ex	0.398***	0.405***	0.149*	1			
P/E	0,045	-0.647***	-0,105	-0.150*	1		
Le/P	-0.467***	-0.243**	0.524***	-0.245***	-0,131	1	
Ex/E	0,079	-0,07	-0,068	0.876***	0.189*	-0.149*	1

See explanations to Table 2.

* P<0.05

**P<0.01

***P<0.001.

**Fig 9:**
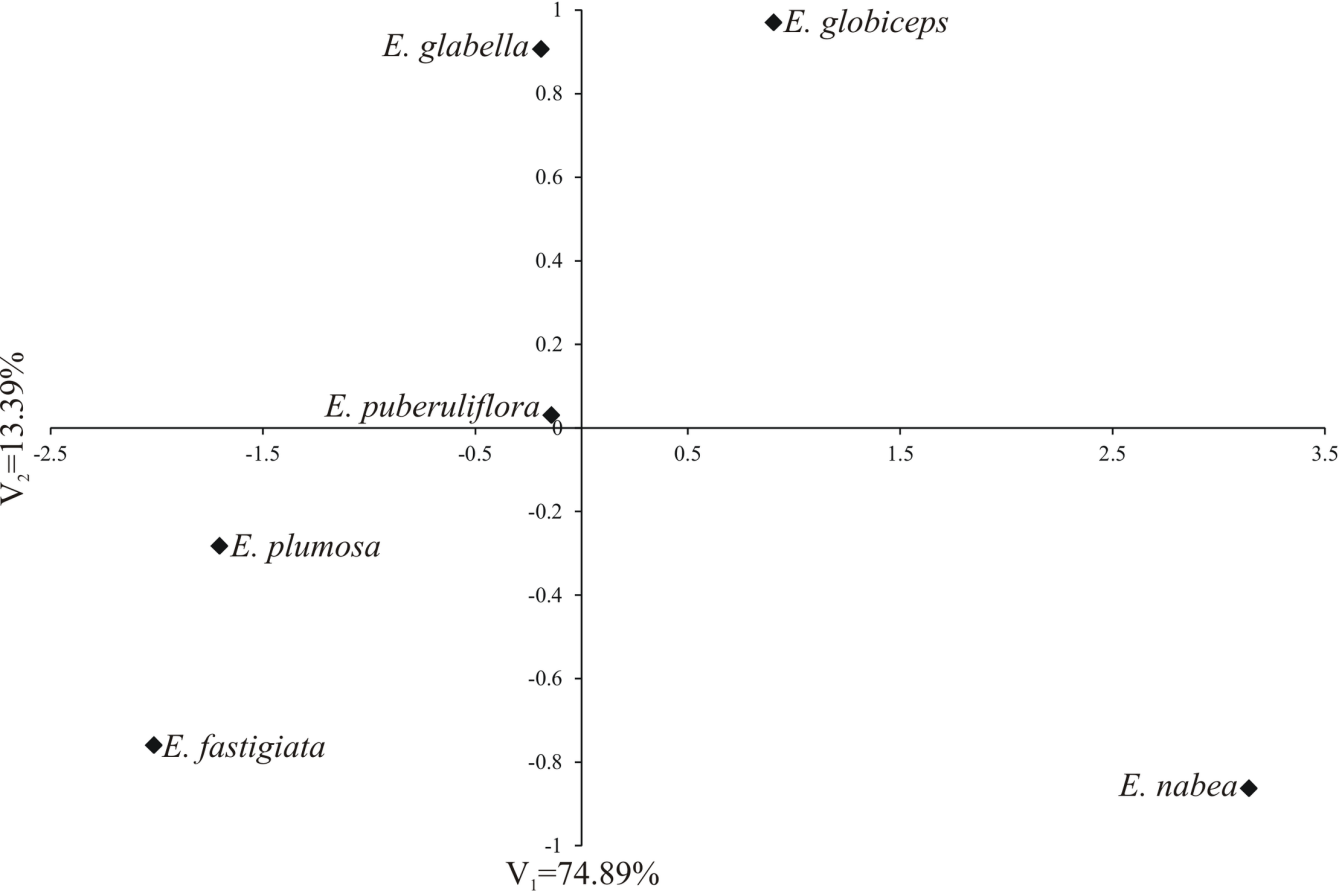
Box-and-whisker diagram of the values of P/E for monads and P/d for tetrads.

**Fig 10:**
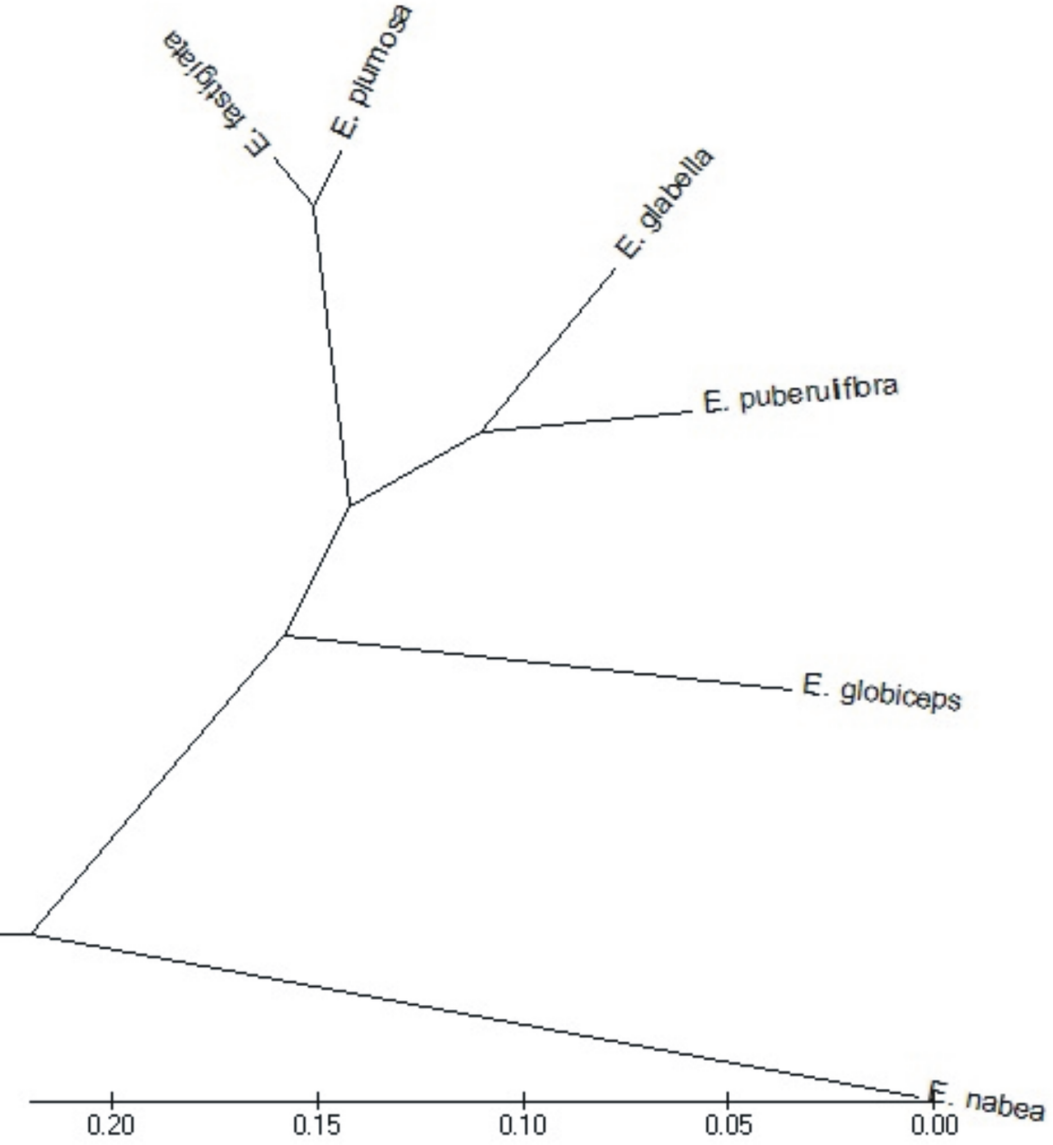
Box-and-whisker diagram of the values of 2f/D, classified by the tetrads’ species.

The greatest variability in all the analysed phenotypic traits expressed jointly with the greatest Mahalanobis distance was observed between the *E*. *fastigiata* and *E*. *nabea* (5.183) as well as between *E*. *nabea* and *E*. *plumosa* (4.887) (Fig 9). In turn, the greatest phenotypic similarity (the smallest Mahalanobis distances) was observed for *E*. *fastigiata* and *E*. *plumosa* (0.808). The first two canonical variables accounted for 88.28% of total multivariate variability between species with monads (Fig 9). This diagram of the first two canonical variables was used to divide the studied species into four groups. The first and second group comprised one taxon *E*. *nabea* and *E*. *globiceps*. The third group embraced two taxa, *E*. *glabella* and *E*. *puberuliflora* and to the last group belonging *E*. *plumosa* and *E*. *fastigiata*. The above data were confirmed by the dendrogram obtained as a result of grouping by using Euclides method (Fig 10).

### Tetrads

The pollen grains of *Erica* studied species were usually tricolporate tetrahedral tetrads. Tetrads are heteropolar, because they have different proximal and distal faces (Figs 2A–2N, 3A–3N, 4A–4R, 5A–5S, 6A–6P, 7A–7P).

All studied traits have a normal distribution as well as a multivariate normality. Results of MANOVA indicate that the species were significant (Wilk’s λ = 0.0008521; F_418,11878_ = 27.00; P < 0.0001) different for all 11 traits. Results of analysis of variance for 11 traits [D (F_38,1129_ = 340.82), d = E (F_38,1129_ = 185.63), P (F_38,1129_ = 102.39), 2f = Le (F_38,1129_ = 202.56), Se (F_38,1129_ = 8.25), Ex (F_38,1129_ = 35.21), D/d (F_38,1129_ = 8.81)], P/d (F_38,1129_ = 16.79)], 2f/D (F_38,1129_ = 28.59)], Ex/Se (F_38,1129_ = 15.00)], Ex/d (F_38,1129_ = 37.23)] at the significance level α = 0.001 (Tables 3–5).

**Fig 11:**
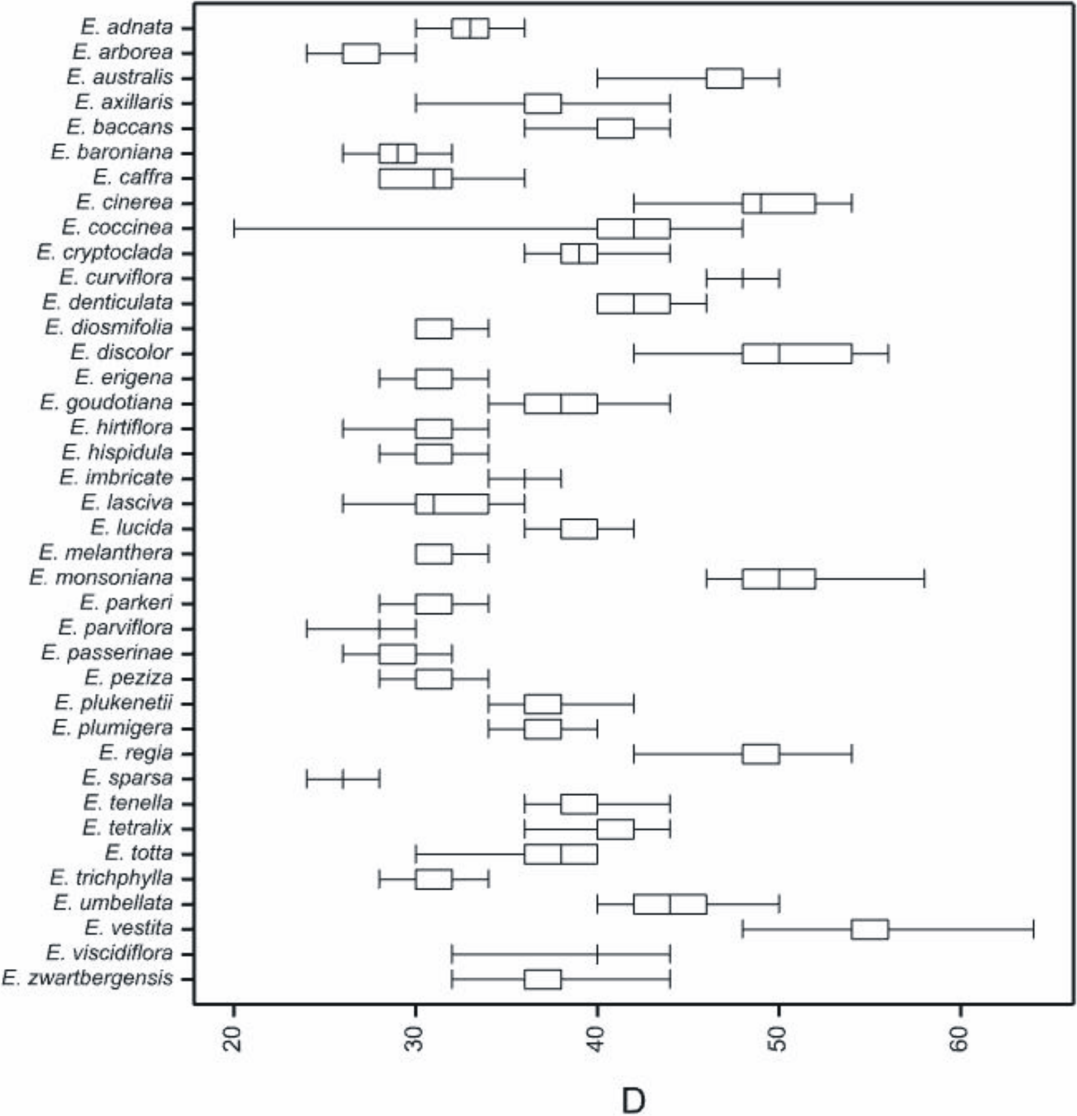
Distribution of 39 *Erica* species with tetrads in the space of two first canonical variables.

The tetrad diameter (D) was 37.37 (20–64) μm. The smallest mean D were found for the pollen of *E*. *sparsa* (26.13), and the largest for *E*. *vestita* (55.07 μm) (Tables 3–5, Fig 11). A majority of small tetrads occurred in the *E*. *sparsa* sample (all measured tetrad were small <30 μm, at a very narrow range of the tetrad diameter; 24–28 μm). It was similar in *E*. *arborea* (24–30 μm). On the other hand, the largest tetrads (all with diameter >47 μm) were found in *E*. *vestita* (48–64 μm). The highest range of the tetrad diameter (20–48 μm) were found in *E*. *coccinea* (Fig 11).

The mean length of the pollen grains equatorial diameter (d) was 25.96 (16–44) μm. The shortest mean equatorial diameter occurred in the pollen of *E*. *sparsa* (18 μm), while the longest was in *E*. *cinerea* and *E*. *vestita* (36.4 μm) (Tables 3–5).

The tetrad diameter (D) is usually greater, than the length of the pollen equatorial diameter (d). D/d ratio was 1.45 (0.77–2.5) and in averange ranged from 1.36 in *E*. *adnata* to 1.66 in *E*. *lucida* (Tables 3–5).

The average length of the polar axis (P) was 19.70 (10–34) μm. Generally speaking, the smallest mean P were found for the pollen of *E*. *erigena* (12.33 μm) and the largest for *E*. *vestita* 29.2 μm; Tables 3–5).

The mean shape (P/d) of pollen grains in tetrad was 0.76 (0.38–1.3) (Fig 8, Tables 3–5). The most flattened pollen grains were found in *E*. *adnata* (0.59) and the most elongated (1.91) in *E*. *plumigera*. Pollen grains of the species examined were most frequently oblate (48,1%) and suboblate (31,1%), rarely oblate-spheroidal (16.3%) and spheroidal (2,3%), peroblate (1.1%), prolate-spheroidal (0.8%) and subprolate (0.4%) pollen were found only sporadically.

Mean exine apocolpial thickness (Ex) is 1.36 (1.0–2.0) μm and septal exine thickness (Se) - 2.83 (2.0–6.0) μm. The apocolpial exine is mostly the half thinner, than the septal exine thickness. The mean Ex/Se ratio is 0.51 and ranged from 0.25 to 1.0. The relative apocolpial exine thickness (Ex/d) was 0.05 (0.02–0.13) (Tables 3–5).

**Fig 12:**
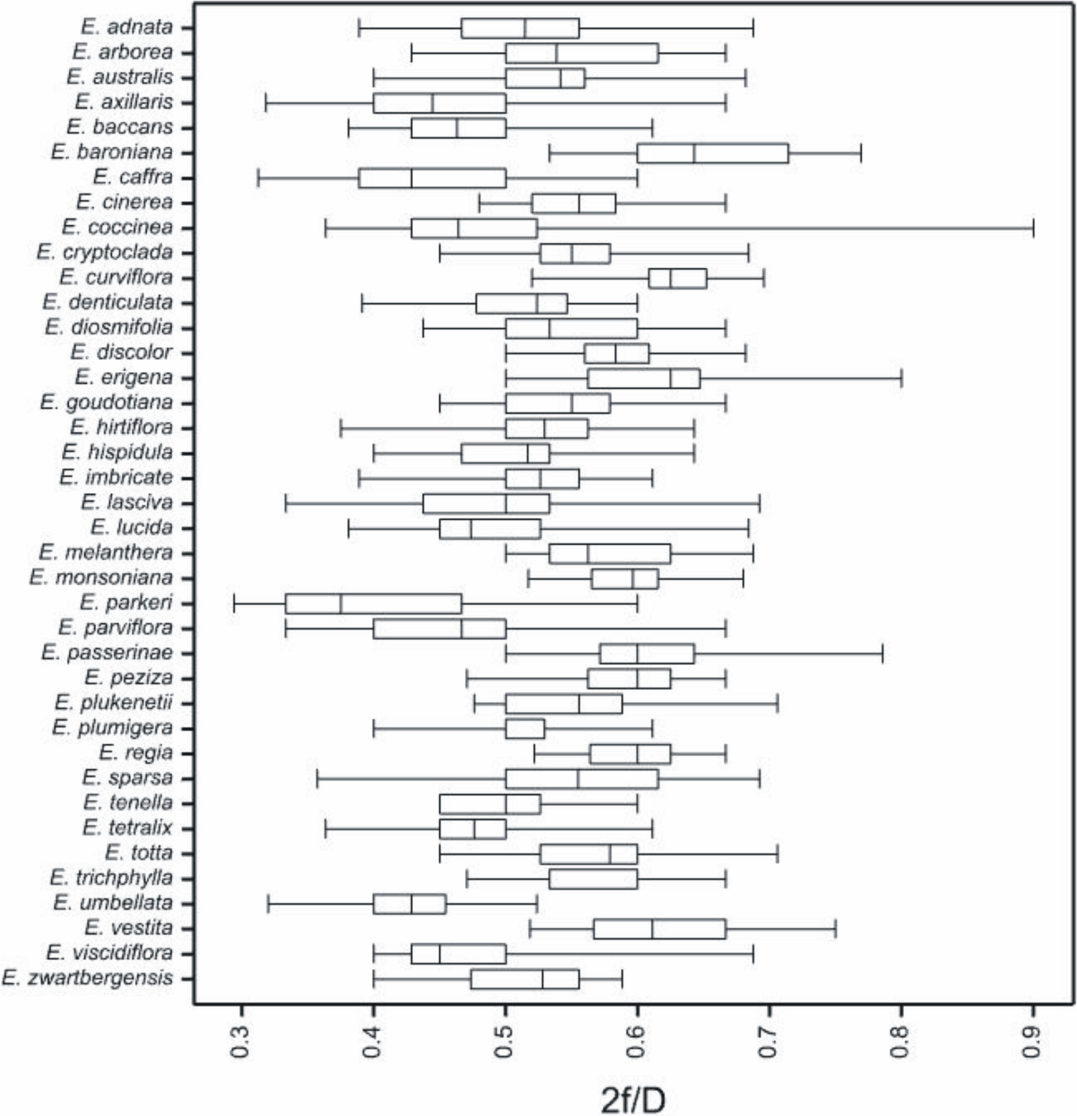
Dendrogram of cluster groupings of *Erica* species with tetrads on the basis of pollen grain morphological features.

The apertures form pairs at six points in the tetrad. Three apertures pairs appear at the proximal and distal face of the tetrad. According to Hesse et al. [37] we have adopted that in *Erica* species occurs colporus, that means compound aperture composed of a colpus (ectoaperture) combined with an endoaperture of variable size and shape. Colpori were arranged regularly, more or less evenly spaced, and were the average length; mean length of colpus (2f) is 19.75 (10.0–42.0) μm (Tables 3–5). On average, the length of colpus (2f) constituted 0.53 of the tetrad diameter (D), which means that colpus is mostly a half shorter than tetrad diameter (Fig 12). This feature (2f/D) wykazuje duży zakres wartości from 0.29 to 0.90. The shortest mean colpi were found in *E*. *parkeri* (12.13 μm) and the longest in *E*. *vestita* (33.87 μm). Colpi were fusiform in outline. Their width was usually slightly greater in the equatorial region. Colpus membrane are psilate to granulate. Endoaperture in colporus were circular or elliptic in outline with irregular margins, with or without costae. Endoaperture are usually located in the middle of colpi, less frequently asymmetrically, usually singly. Opened, single endoapertures occurred in the majority of studied species. In some species (*E*. *cinerea*, *E*. *coccinea*, *E*. *denticulata*, *E*. *hirtiflora*, *E*. *regia*, *E*. *tenella*, *E*. *tetralix*) a bridge is also present, understood according to Hesse et al. (2009), as an exine connection between the margins of a colpus in the equatorial region or as an exine connections within tetrads.

In SEM exine ornamentation is usually granulate and granulate-fossulate, rarely fossulate or psilate (see: pollen key). In many species microgranules with diameters from 0.1 to 0.3 μm were found, while in *E*. *caffra* there is only a very fine microgranules with diameters 0.1 μm. Their number varies from a few to very numerous. Some *Erica* studied species create a specific exine ornamentation, that occurs mainly in tetrads (e.g. *E*. *curviflora*, *E*. *discolor*, *E*. *erigena* or *E*. *tetralix*) very rarely in monads (*E*. *fastigiata*). On the polar areas (in monad) and at the proximal poles of the tetrad and along colpi exine were psilate, and in the interapertural areas granulate, granulate-fossulate or fossulate.

The performed correlation analysis indicates statistically significant correlation coefficients of 23 out of 55 coefficients (Table 7). The positive correlation coefficients we observed for pair of tratis: D and d = E, D and P, D and 2f = Le, D and Se, d = E and P, d = E and 2f = Le, P and 2f = Le, P and Se, P and P/d, 2f = Le and 2f/D, Se and Ex, Ex and D/d, Ex and P/d, Ex and Ex/Se, Ex and Ex/d, D/d and P/d, D/d and Ex/Se, D/d and Ex/d, P/d and Ex/d, Ex/Se and Ex/d. The negative coefficients was observed for: D and Ex/d, d = E and Ex/d, 2f = Le and Ex/d (Table 7).

**Table 7:** Correlation matrix for the observed features of species with tetrads.

	D	d = E	P	2f = Le	Se	Ex	D/d	P/d	2f/D	Ex/Se	Ex/d
D	1										
d = E	0.973***	1									
P	0.923***	0.909***	1								
2f = Le	0.897***	0.884***	0.801***	1							
Se	0.329*	0,299	0.386*	0,273	1						
Ex	0,18	0,084	0,248	0,077	0.364*	1					
D/d	0,283	0,06	0,21	0,196	0,131	0.446**	1				
P/d	0,118	0,032	0.441**	0,008	0,255	0.426**	0.365-	1			
2f/D	0,146	0,158	0,071	0.561***	-0,037	-0,132	-0,04	-0,189	1		
Ex/Se	0,003	-0,081	0,036	-0,076	-0,161	0.852***	0.405*	0,295	-0,12	1	
Ex/d	-0.398*	-0.492**	-0,305	-0.414**	0,076	0.812***	0.352*	0.346*	-0,158	0.823***	1

See explanations to Table 3.

* P<0.05

** P<0.01

*** P<0.001

**Fig 13:**
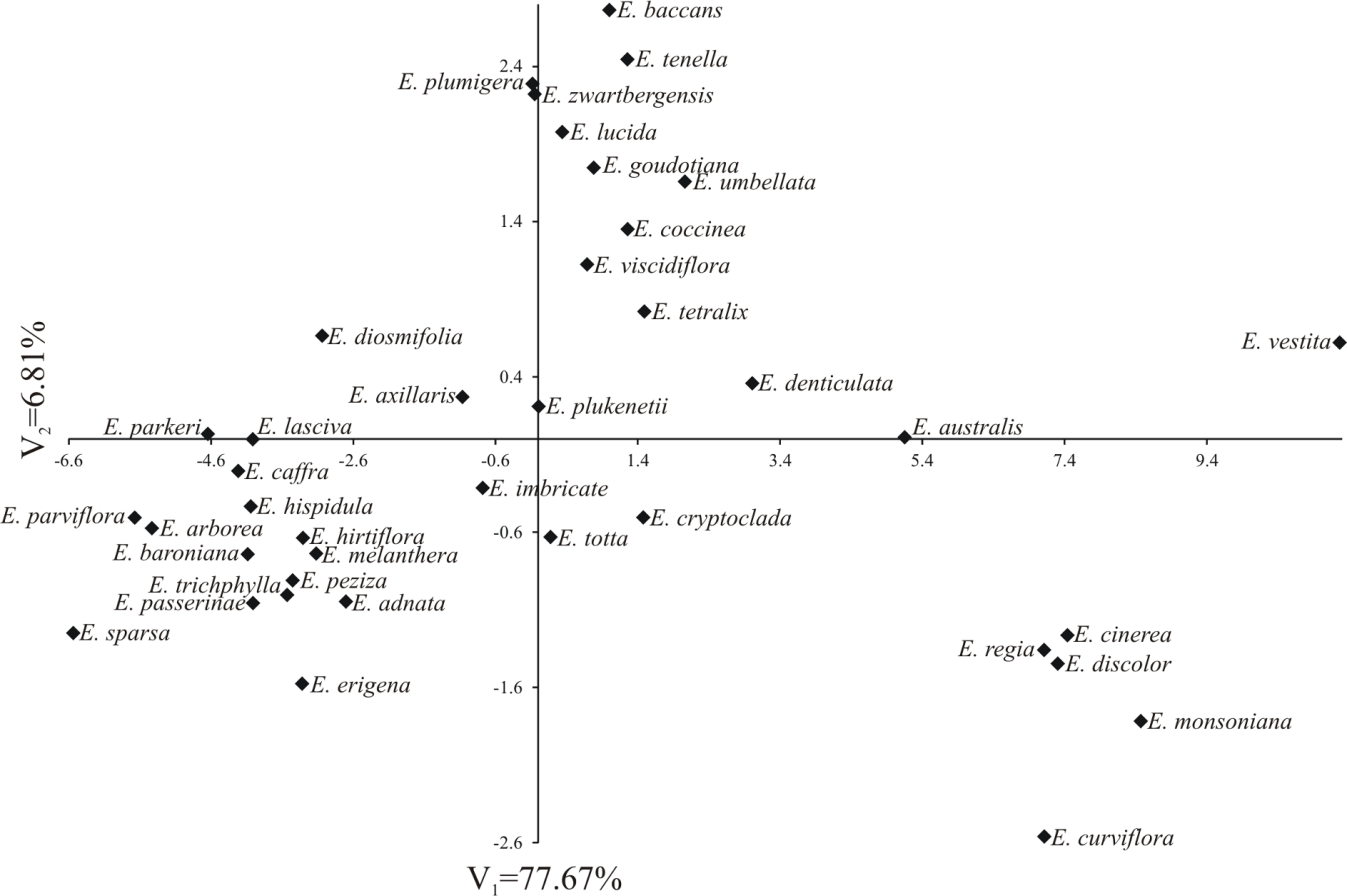
Distribution of six *Erica* species with monads in the space of two first canonical variables.

The first two canonical variables accounted for 84.48% of total multivariate variability between species (Fig 13). The greatest variability in terms of all the analysed traits expressed jointly with the greatest Mahalanobis distance was recorded for the *E*. *vestita* and *E*. *sparsa* (18.298). In turn, the greatest phenotypic similarity was observed for *E*. *plumigera* and *E*. *zwartbergensis* (0.675), *E*. *peziza* and *E*. *trichophylla* (0.930) as well as for *E*. *hirtiflora* and *E*. *melanthera* (0.956).

**Fig 14:**
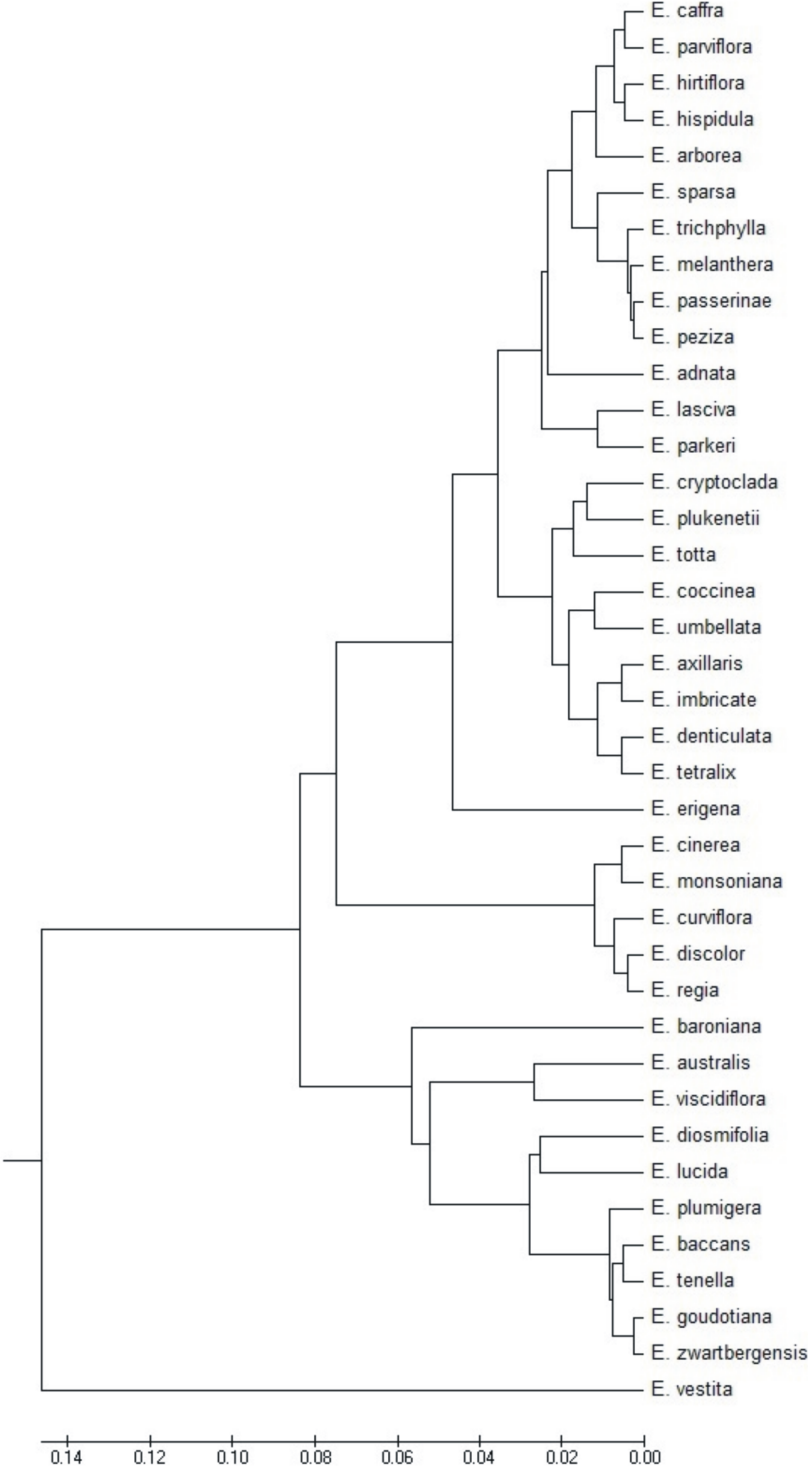
Dendrogram of cluster groupings of *Erica* species with monads on the basis of pollen grain morphological features.

The above data were confirmed by the dendrogram obtained as a result of agglomeration grouping using Euclides method (Fig 14). The studied species were divide to into three groups. The first group comprised one taxon *E*. *vestita*, which is most different from all other species. To the second group belonging ten species, from which the most distinctive pollen grains features shows *E*. *baroniana*. The third, large group embraced 28 species. *E*. *regia*, *E*. *discolor* and *E*. *curviflora* and *E*. *cinerea* and *E*. *monsoniana* are similar to each other and differ from the rest of the group. *E*. *erigena* occupies also a separate position in the third group. Other species from this group fall into the seven non-significant subgroups.

From 39 analysed *Erica* species creating tetrads, on the basis of the tetrad diameter and exine ornamentation eight species (*E*. *arborea*, *E*. *australis*, *E*. *cinerea*, *E*. *erigena*, *E*. *parviflora*, *E*. *passerinae*, *E*. *regia*, *E*. *tetralix*) were separated and other heaths created a small groups, usually containing two or three species, rarely up to a seven species (see: Pollen key).

### Pollen key

1 Monads. Pollen grains elongated P/E 1.24 (0.93–1.63).............................................2
1*Tetrads. Pollen grains flattened P/d = E 0.76 (0.38–1.30)............................................6
2 Pollen small, on average P ≤25 μm)...................................................................3
2* Pollen medium, on average P >25 μm)..............................................................5
3 Exine ornamentation granulate-fossulate.......................................... *E*. *puberuliflora*
3* Exine ornamentation psilate with microgranules......................................................4
4 Microgranules numerous (diameter 0.1–0.3 μm). Pollen small (97% in sample)..............................................................................................*E*. *nabea*
4* Microgranules very numerous (diameter 0.1μm). Pollen small (50% in sample) or medium.....................................................................................................................*E*. *globiceps*
5 Exine ornamentation psilate with microgranules.................................................*E*. *glabella* 5* Exine ornamentation granulate, at the poles and along the colpi psilate...........*E*. *fastigiata*
5** Exine ornamentation granulate-fossulate............................................. *E*. *plumosa*
6 Exine ornamentation psilate with microgranules..............*E*. *caffra*, *E*. *lanipes*, *E*. *plukenetii*
6* Exine ornamentation granulate.......................................................................7
6** Exine ornamentation granulate-fossulate...........................................................9
6*** Exine ornamentation fossulate....................................................................17
7 With microgranules.......................................................................................8
7* Without microgranules..................................*E*. *axillaris*, *E*. *baccans*, *E*. *diosmifolia*
8 Tetrad large, mean diameter >47 μm.........................................................................*E*. *cinerea*
8* Tetrad medium, mean diameter 31–46 μm.................................*E*. *adnata*, *E*. *denticulata*
9 Exine ornamentation granulate-fossulate, psilate at the poles and along the colpi.............10
9* Exine ornamentation granulate-fossulate, not psilate at the poles and along the colpi......13
10 With microgranules.....................................................................................11
10* Without microgranules..............................................................................12
11 Tetrad large with a few microgranules..........................................................*E*. *regia*
11*Tetrad small, mean diameter <30 μm with very numerous microgranules.......*E*. *passerinae*
12 Tetrad large, mean tetrad diameter >47 μm.......................................................*E*. *curviflora*, *E*. *discolor*, *E*. *monsoniana*, *E*. *vestita*
12* Tetrad medium sized, mean tetrad diameter 31–46 μm...................................*E*. *erigena*
13 With microgranules...................................................................................14 13* Without microgranules...............................................................................16
14 Microgranules very clearly visible and very numerous..........................................15
14* Microgranules not so clearly visible, but numerous................................*E*. *baroniana*, *E*. *hirtiflora*, *E*. *lucida*, *E*. *melanthera*, *E*. *umbellata*
15 Tetrad small...................................................................................*E*. *parviflora*
15* Tetrad medium.............................................*E*. *hispidula*, *E*. *imbricate*, *E*. *parkeri*
16 Tetrad large.................................................................................*E*. *australis*
16* Tetrad medium sized.................................................................*E*. *cryptoclada*, *E*. *goudotiana*, *E*. *peziza*, *E*. *plumigera*, *E*. *tenella*, *E*. *totta*, *E*. *trichphylla*
16** Tetrad small.............................................................................. *E*. *arborea*
17 Exine ornamentation fossulate, at the poles and along the colpi psilate with very numerous microgranules..................................................................................... *E*. *tetralix*
17*Exine ornamentation fossulate, not psilate at the poles and along the colpi..................18
18 With very numerous microgranules................................. *E*. *sparsa*, *E*. *zwartbergensis*
18* With a few microgranules...................................................*E*. *coccinea*, *E*. *lasciva*

## Discussion

The research results presented here corroborate the opinions of other palynologists (e.g. [6, 26, 28, 35, 36, 38, 39, 50]) that the diagnostic features of *Erica* species pollen grains comprise: the dispersal unit of the pollen grains, pollen and tetrad size, pollen shape (P/E and P/d ratios) and exine ornamentation.

All researchers agree that the dispersal unit is one of the most important features of the pollen grains of the *Erica* genus. On this basis, *Erica* species can be divided into two groups which have monads and tetrads. They were analysed [51] 138 *Erica* species from southern Africa, 125 of which had tetrads and only 13 had monads. Similar proportions were observed in 21 European *Erica* species of which only *E*. *terminalis* and *E*. *spiculifolia* (= *Bruckenthalia spiculifolia*) had pollen in monads [6, 20, 22]. In addition, in the present study, species with tetrads (39) dominated over those with monads (6). On the other hand, in the minor genera, the majority of the species studied had monads (57) and the remaining ones (30) had tetrads [6].

One of the palynologists [28] reported an interesting result concerning the dispersal unit of the pollen grains of the studied genus. In his opinion, *E*. *glabella* created monad and tetrad pollen grains. This may be because of that, the pollen grains of *E*. *glabella* are loosely grouped together in tetrads, which are separated easily into monads. Especially, that monad pollen grains of *Erica* are the most advanced pollen character state and derived from tetrads. Although other [26] claims that no species has been found with monad and tetrad pollen grains in the *Erica* as well as other genera of the family *Ericaceae*. In some studies [6], pollen of *E*. *glabella* were described as monad, but in [28] this pollen was described as tetrad. The results of the present study did not corroborate the results cited above [28]: the pollen grains of *E*. *glabella* were described here as monad. Furthermore, all the remaining *Erica* species under investigation in this study had either monad or tetrad pollen grains, which would confirm the hypothesis quoted above [26]. However, inside one section, e.g. *Callista*, both species-forming monads (*E*. *fastigiata*) as well as tetrads (*E*. *denticulata*) can be observed. Perhaps this interesting problem will be clarified through further palynological investigations of the large and diverse *Erica* genus.

The mean measurement results for the monads and tetrads were, in general, consistent with those reported by the above-mentioned palynologists, although higher value ranges were generally obtained for the examined features [e.g. tetrad diameter (D)—result from present study 20–64 μm, [35] 25.6–47 μm, [28] and [36] the same results 29.8–48.4 μm; polar axis (P)—result from present study 10–34 μm, [28] 15.5–26.4 μm)]. This may be due to the examination of different species and larger pollen samples.

The present study fully confirmed the hypothesis presented in [36] that the P/E ratio shows distinct differences between the *Erica* monads and tetrads. The pollen grains are usually elongated in monads and flattened in tetrads. However, the study presented here does not support the view of the above-quoted researchers that exine ornamentation allows a distinction between *Erica* monads and tetrads because in the current case, in both of these dispersal units, similar exine ornamentation types occurred.

In addition, this study failed to corroborate the importance of the 2f/D ratio (length of ectoaperture to tetrad diameter) on the basis of which two authors [36] distinguished two distinctly different palynomorphological *Erica* species groups, which is somewhat surprising since their range for this feature amounted to 0.43–0.75, smaller than that obtained in the present study (0.29–0.90). It is worth stressing that the mean values of this feature in individual species present a small range (0.39–0.64) which does not allow identification of species groups. A similar situation occurs in the case of the L/P ratio (length of ectoaperture to length of polar axis). The species producing monads analysed in the present study exhibited a narrow range of mean values for this feature (0.67–0.85), therefore, it was not possible to distinguish species groups. That is why, identification among the same species of two distinct groups with an almost identical range of the described feature (0.68–0.84) raises some doubts [36].

The greatest differences in descriptions of the pollen grains of *Erica* species concerned the exine ornamentation. These resulted from the high variability of exine ornamentation types and subtypes in individual *Erica* species, as well as from simplified or expanded classifications of features [26, 28, 35, 36]. In the present study, it was possible to distinguish the greatest number of *Erica* species or species groups on the basis of exine ornamentation traits (see: Pollen key). The present study supports the opinions of other authors [6, 26, 36] in that exine ornamentation in the *Erica* species widely differed but was commonly characterised by numerous microgranules. It was exactly the presence or absence, and number of microgranules and exine ornamentation type which proved to be vital in the diagnosis of the *Erica* species under investigation. The same types of exine ornamentation was found in species with tetrads and monads [26]. The results from the present study were similar; boundaries between the exine ornamentation types in the tetrads and monads were not distinct. In the analysed monads, psilate, granulate, granulate-psilate or granulate-fossulate exine ornamentation occurred, while in the tetrads–apart from those mentioned above–fossulate ornamentation also occurred. In comparison with the present study, in the case of the species examined by two palynologists [28, 36], a greater variability of exine ornamentation types was observed. Rugulate, verrucate, striate, rugulate-psilate and gemmate-psilate pollen grains were reported. This can certainly be associated with the greater variability in this feature noted in the *Erica* species in their research, as well as with a more expanded classification of exine ornamentation types than that adopted in the present study, follows [37–39].

Very interesting results were obtained following the statistical analysis of the biometrical features of the pollen in the species producing tetrads (Fig 12, Table 1), but these results are not unequivocal. Despite the heterogeneity of the species groups distinguished, they often included, although not always, species belonging to the same subgenera or sections distinguished in accordance with the adopted systematic division of the *Erica* genus (Fig 12, Table 1). Species from the *Syringodea* subgenus and *Gigandra* (*E*. *coccinea* and *E*. *plukenetii)* and *Evanthe* (*E*. *curviflora E*. *discolor*) sections grouped together, whereas species from the *Pleurocallis* (*E*. *regia*, *E*. *vestita*) section occupied a separate position. The most numerous subgenus—*Euerica*, was represented by 14 species belonging mostly to group 3, and less frequently to group 2. Very similar features were found to occur in species of pollen grains which belonged to *Ephebus* (*E*. *caffra*, *E*. *parviflora*, *E*. *hirtiflora*, *E*. *peziza*) and *Arsace (E*. *arborea*, *E*. *hispidula*) sections, as well as *E*. *trichophylla* derived from the *Orophanes* section. The remaining species differed from one another to a varying degree but the most similar pollen grains were found in *E*. *tetralix* and *E*. *umbellate*. On the dendrogram, the representatives of the *Eurica* subgenus which differed from the others the most were *E*. *tenella* and *E*. *zwartbergensis*, whose pollens were very similar to each other. Finally, four heathers from Madagascar occurred in group 2 (*E*. *baroniana*, *E*. *goudotiana*) and group 3 (*E*. *cryptoclada*, *E*. *parkeri*), with the second pair characterised by very similar pollen grains (Fig 12, Table 1). From among the *Chlamydanthe* subgenus, three species clustered together, two derived from the *Trigemma* (*E*. *plumigera*, *E*. *baccans*) section and one from the *Elytrostegia* (*E*. *diosmifolia*) section; the remaining heathers were found in other groups. In the *Platystoma* subgenus, similar features were found to occur in four (*E*. *melanthera*, *E*. *passerinae*—section *Gamochlamys*, *E*. *sparsa*—section *Polycodon* and *E*. *adnate*—section *Eurystoma*) out of five of the species examined. Dissimilarity was observed in *E*. *lucida* from section *Eurystoma*. From among the minor genera species, only two formed tetrads, namely *E*. *axillaris* and *E*. *totta*, which belonged to the same group.

In the case of species producing monads, the results obtained as well as the distribution of taxons acquired on their basis failed to reflect the internal division of the *Erica* genus generally accepted in taxonomy (Fig 14, Table 1). The species producing monads did not create a homogeneous group in the system. Four of them (*E*. *glabella*, *E*. *globiceps*, *E*. *plumosa*, *E*. *puberuliflora*) came to the genus *Erica* from minor genera. They were included in different groups, with the exception of *E*. *glabella* and *E*. *puberuliflora* (Fig 14). The pollen grains of *E*. *fastigiata* were most similar to *E*. *plumosa*, although the former species derives from the section *Callista*, to which *E*. *denticulata* producing tetrads also belongs, whereas the latter species belongs to the minor genera. Only *E*. *nabea* exhibited discreteness, and belongs to a separate *Chlamydanthe* subgenus and *Adelopetalum* section.

## Conclusions

The study revealed that the diagnostic features of the pollen grains studied were: pollen dispersal unit, exine ornamentation, P/E ratio, tetrad diameter (D) and length of polar axis (P). On the basis of these traits, 14 *Erica* species (six creating monads and eight—tetrads) were distinguished which, in the case of pollen features, constitutes a significant number. Other heaths created small groups, usually containing two or three species, but up to seven species. Our study, based on the highest number of *Erica* species (45) analysed so far, corroborated the view that an examination of palynological features may assist in clarifying classification systems for the large and taxonomically very difficult *Erica* genus, in particular, at the level of the subgenus and section, but also at species level. The results obtained indicate the need to continue palynological investigations on the *Erica* genus.
